# Supplementary material for: A nanopore interface for higher bandwidth DNA computing
Source: Nat Commun. 2022 Aug 20;13:4904. doi: 10.1038/s41467-022-32526-3 (PMC9392746; doi:10.1038/s41467-022-32526-3)
Supplement: Supplementary file 1 — Supplementary Information [file 41467_2022_32526_MOESM1_ESM.pdf]

Supplementary Materials for

A nanopore interface for higher bandwidth DNA computing

Karen Zhang, Yuan-Jyue Chen, Delaney Wilde, Kathryn Doroschak, Karin Strauss, Luis Ceze, Georg Seelig, and Jeff Nivala

Tables 1-4

Figures 1-19

Circuit #	Circuit Component	Sequence	Purification Method
0	Gate	AGAGTGTGGAGTTGATAGGAGAG	Standard Desalting
	Output	ACACCTTACTCTCTACTCTCCTATCAACTCCACATTTTTTNNNNNNTT/3BioTEG/	HPLC
	Input	CCTATCAACTCCACACTCTCACTAATTCTACATC	Standard Desalting
	Fuel	TACCTCATTCAAACTCTCTCCTATCAACTCCACA	Standard Desalting
	Quencher	/5IAbRQ/ACACCTTACTCTCTA	HPLC
	Fluorophore	AGAGTAGAGAGTAAGGTGT/36-FAM/	HPLC
1	Gate	AGAGAATAATGGTTGTAGGAGAG	Standard Desalting
	Output	CTTCTTATACCACACCTCTCCTACAACCATTATTTTTTTNNNNNNCA/3BioTEG/	HPLC
	Input	CCTACAACCATTATTCTCTCAATCTACCAAACTC	Standard Desalting
	Fuel	ACAAATACCTCATCCCTCTCCTACAACCATTATT	Standard Desalting
	Quencher	/5IAbRQ/CTTCTTATACCACAC	HPLC
	Fluorophore	AGAGGTGTGGTATAAGAAG/36-FAM/	HPLC
2	Gate	AGAGTAAGTATAGAGGTGAAGAG	Standard Desalting
	Output	CAACTACAATCTCTCCTCTTCACCTCTATACTTATTTTTTNNNNNNTA/3BioTEG/	HPLC
	Input	TCACCTCTATACTTACTCTTCCTTCATCTTCTAC	Standard Desalting
	Fuel	TCTCCAATTTCAACTCTCTTCACCTCTATACTTA	Standard Desalting

	Quencher	/5IAbRQ/CAACTACAATCTCTC	HPLC
	Fluorophore	AGAGGAGAGATTGTAGTTG/36-FAM/	HPLC
3	Gate	AGAGAGGATTAGGATAGTGAGAG	Standard Desalting
	Output	AACCACATTAACCTTCTCTCACTATCCTAATCCTTTTTTTNNNNNNCC/3BioTEG/	HPLC
	Input	CACTATCCTAATCCTCTCTAAACCTTACCACCAC	Standard Desalting
	Fuel	CCTTCTCAACTCCTCCTCTCACTATCCTAATCCT	Standard Desalting
	Quencher	/5IAbRQ/AACCACATTAACCTT	HPLC
	Fluorophore	AGAGAAGGTTAATGTGGTT/36-FAM/	HPLC
4	Gate	AGAGGGTGTTTAGAGTTTAAGAG	Standard Desalting
	Output	TTATCCAACTCACTACTCTTAAACTCTAAACACCTTTTTTNNNNNNAT/3BioTEG/	HPLC
	Input	TAAACTCTAAACACCCTCTAATAACACCTCCTAA	Standard Desalting
	Fuel	CTCTTCTTTCCAAACCTCTTAAACTCTAAACACC	Standard Desalting
	Quencher	/5IAbRQ/TTATCCAACTCACTA	HPLC
	Fluorophore	AGAGTAGTGAGTTGGATAA/36-FAM/	HPLC
5	Gate	AGAGAAAGTGATAAGATGGAGAG	Standard Desalting
	Output	TCTTTCACCTCACATCTCTCCATCTTATCACTTTTTTTTNNNNNNCA/3BioTEG/	HPLC
	Input	CCATCTTATCACTTTCTCTATTACTTCCTACACC	Standard Desalting

	Fuel	ATCCTCCTTCCATCCCTCTCCATCTTATCACT TT	Standard Desalting
	Quencher	/5IAbRQ/TCTTTCACCTCACAT	HPLC
	Fluorophore	AGAGATGTGAGGTGAAAGA/3Cy5Sp/	HPLC
6	Gate	AGAGGGTATTAGTTAGGTAAGAG	Standard Desalting
	Output	TCCATTTCATTTCACCTCTTACCTAACTAATA CCTTTTTTNNNNNNAA/3BioTEG/	HPLC
	Input	TACCTAACTAATACCCTCTCTCCATAACATT CCA	Standard Desalting
	Fuel	ACTTCTAACAACTACCTCTTACCTAACTAAT ACC	Standard Desalting
	Quencher	/5IAbRQ/TCCATTTCATTTCAC	HPLC
	Fluorophore	AGAGGTGAAATGAAATGGA/36-FAM/	HPLC
7	Gate	AGAGTGTTAGTAGTAGAGTAGAG	Standard Desalting
	Output	AAATTCTATCCACTCCTCTACTCTACTACTAA CATTTTTTNNNNNNCC/3BioTEG/	HPLC
	Input	ACTCTACTACTAACACTCTTCTACATCCACA TCT	Standard Desalting
	Fuel	CACTCAATAACTACCCTCTACTCTACTACTA ACA	Standard Desalting
	Quencher	/5IAbRQ/AAATTCTATCCACTC	HPLC
	Fluorophore	AGAGGAGTGGATAGAATTT/36-FAM/	HPLC
8	Gate	AGAGGTATAAAGGAGTTTGAGAG	Standard Desalting
	Output	TAACTCTACCACAAACTCTCAAACTCCTTTA TACTTTTTTNNNNNNTA/3BioTEG/	HPLC

	Input	CAAACTCCTTTATACCTCTCTCTACTCATCTT CC	Standard Desalting
	Fuel	CCACCTCCATCTATACTCTCAAACTCCTTTAT AC	Standard Desalting
	Quencher	/5IAbRQ/TAACTCTACCACAAA	HPLC
	Fluorophore	AGAGTTTGTGGTAGAGTTA/3Cy5Sp/	HPLC
9	Gate	AGAGTGGTAAGGTAGTTAAAGAG	Standard Desalting
	Output	CTAACAAACTTTACCCTCTTTAACTACCTTAC CATTTTTTNNNNNNTC/3BioTEG/	HPLC
	Input	TTAACTACCTTACCACTCTACATTCCTTCTAA TC	Standard Desalting
	Fuel	ATTTACATCTCAACCCTCTTTAACTACCTTAC CA	Standard Desalting
	Quencher	/5IAbRQ/CTAACAAACTTTACC	HPLC
	Fluorophore	AGAGGGTAAAGTTTGTTAG/3Cy5Sp/	HPLC

Supplementary Table 1: Table of seesaw gate, output strand, input strand, fuel strand, quencher-labeled strand, and fluorophore-labeled strand sequences for all ten circuits used in kinetics analysis and multiplexing experiments. Iowa Black RQ from IDT is used as the quencher molecule and either 6-FAM or Cy 5 is used as the fluorophore in the fluorescent reporter complexes. The nanopore barcode region (red) can be substituted with any barcode from Supplementary Table 2.

Barcode #	Set A	Set B	Set C
0	CAAATA	GGGTTC	/iSpC3/CATAC
1	TCATAC	TGATTG	T/iSpC3/ATAC
2	ATATCT	AGAGTT	TC/iSpC3/TAC
3	CTCCAC	AGAGGA	TCA/iSpC3/AC
4	ATCTAA	ATATCA	TCAT/iSpC3/C
5	CTCAAA	TTCTGT	TCATA/iSpC3/
6	AAATAC	AGCCTC	/iSpC3/CATA/iSpC3/
7	TCCAAC	GATACT	T/iSpC3/AT/iSpC3/C
8	CAAAAC	TCTCTG	TC/iSpC3//iSpC3/AC
9	ACCTCC	AATCAA	T/iSpC3//iSpC3/TAC
10	-	TGGAAG	TCA/iSpC3//iSpC3/C
11	-	GCACAT	TCAT/iSpC3//iSpC3/
12	-	-	T/iSpC3//iSpC3//iSpC3/AC
13	-	-	AA/iSpC3/CAA

Supplementary Table 2: Table of all explored output strand barcodes organized into Set A (randomly selected), Set B (based on predictive model) and Set C (contains abasic sites). Abasic sites are denoted as /iSpC3/ (C3 Spacer phosphoramidite modification from IDT) .

Circuit Component	Sequence	Purification Method
Gate	TGAGTGTGATTGTGTTATGAGTG	Standard Desalting
Output	CAACATATCAATTCACTCATAACACAATCACATTTTTTTCATACCA/3BioTEG/	HPLC
Input	CATAACACAATCACACTCACCACCAAACTTCA	Standard Desalting
Fuel	CACTAACATACAACACTCATAACACAATCACA	Standard Desalting
Quencher	/5IAbRQ/CAACATATCAATTCA	HPLC
Fluorophore	TGAGTGAATTGATATGTTG/3Cy5Sp/	HPLC

Supplementary Table 3: Table of seesaw gate, output strand, input strand, fuel strand, quencher-labeled strand, and fluorophore-labeled strand sequences for clamped circuit from Supplementary Figure 1. Iowa Black RQ from IDT is used as the quencher molecule and Cy 5 is used as the fluorophore in the reporter complex. Barcode A1 (red) from Supplementary Table 2 was used in this circuit's output strand.

**Probe Component**	**Sequence**	**Purification Method**
**let-7a Probe**		
Input	UGAGGUAGUAGGUUGUAUAGUU	HPLC
Helper	GAGTGTAGTGGAAGTTGGAG	PAGE
Bottom	AACTATACAACCTACTACCTCACTCCAACTTCCACTACACTC/36-FAM/	HPLC
Top	/5IAbRQ/GAGTGTAGTGGAAGT	HPLC
Output	TGGAGTGAGGTAGTAGGTTTTTTTTTGGAAGTT/3BioTEG/	HPLC
**let-7c Probe**		
Input	UGAGGUAGUAGGUUGUAUGG	HPLC
Helper	TGAGGTATGGAGTGAGTGGA	PAGE
Bottom	AACCATACAACCTACTACCTCATCCACTCACTCCATACCTCA/36-FAM/	HPLC
Top	/5IAbRQ/TGAGGTATGGAGTGA	HPLC
Output	GTGGATGAGGTAGTAGGTTTTTTTTAA/iSpC3/CAATT/3BioTEG/	HPLC
**let-7e Probe**		
Input	UGAGGUAGGAGGUUGUAUAGUU	HPLC
Helper	TGAGGTATGGAGTGAGTGGA	PAGE
Bottom	AACTATACAACCTCCTACCTCATCCACTCACTCCATACCTCA/3Cy5Sp/	HPLC
Top	/5IAbRQ/TGAGGTATGGAGTGA	HPLC
Output	GTGGATGAGGTAGGAGGTTTTTTTTT/iSpC3//iSpC3//iSpC3/ACTT/3BioTEG/	HPLC

Supplementary Table 4: Table of let-7 miRNA input, helper strand, bottom strand (with fluorophore), top strand (with quencher), and output strand (with barcode) for the two-step let-7

detection probes. The barcode region is represented in red. Barcodes B10, C13, and C12 from Supplementary Table 2 were used for the let-7a, let-7c, and let-7e probes respectively.

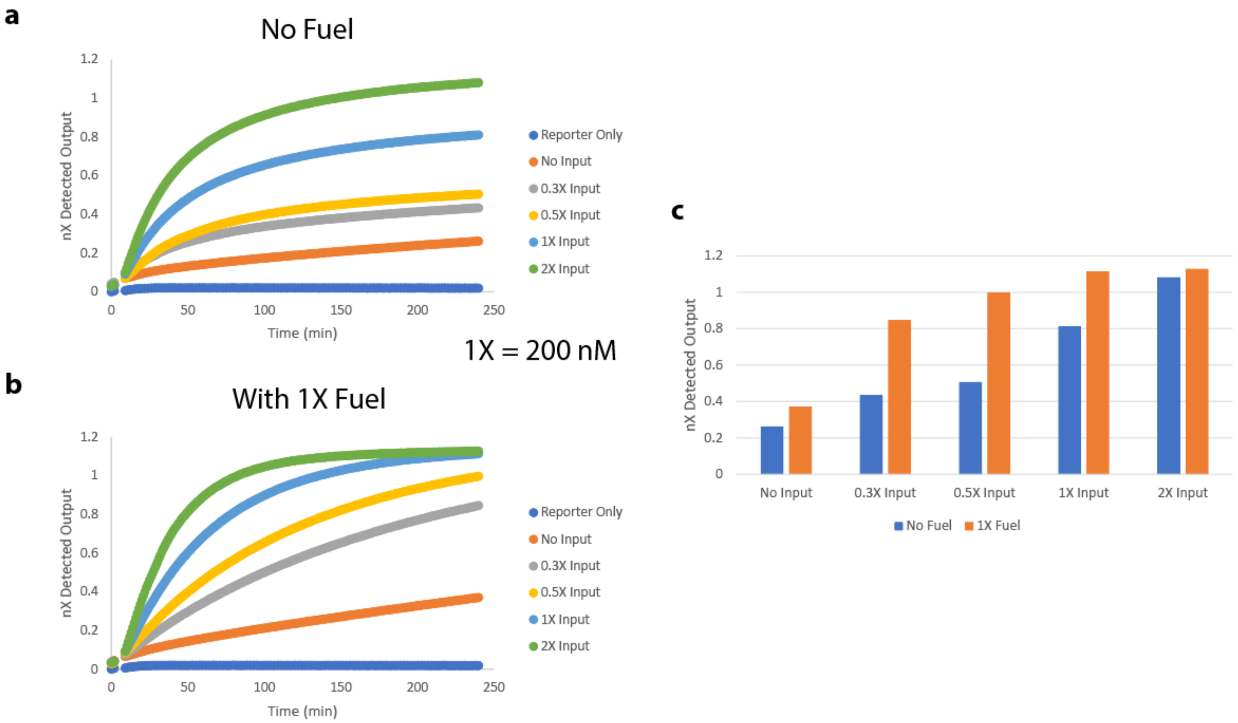

Supplementary Figure 1: Kinetics of a DSD circuit (Circuit 4 with Barcode C8) as measured by a plate reader spectrometer. Circuit was run with a gate complex concentration at 200 nM (= 1X), reporter complex at 6X, streptavidin at 4X, and varying levels of input strand concentrations. This was done **a)** without fuel strand and **b)** with 1X fuel strand. **c)** A comparison of conversion efficiency between the No Fuel and 1X Fuel samples at 240 min is shown in terms of nX detected output.

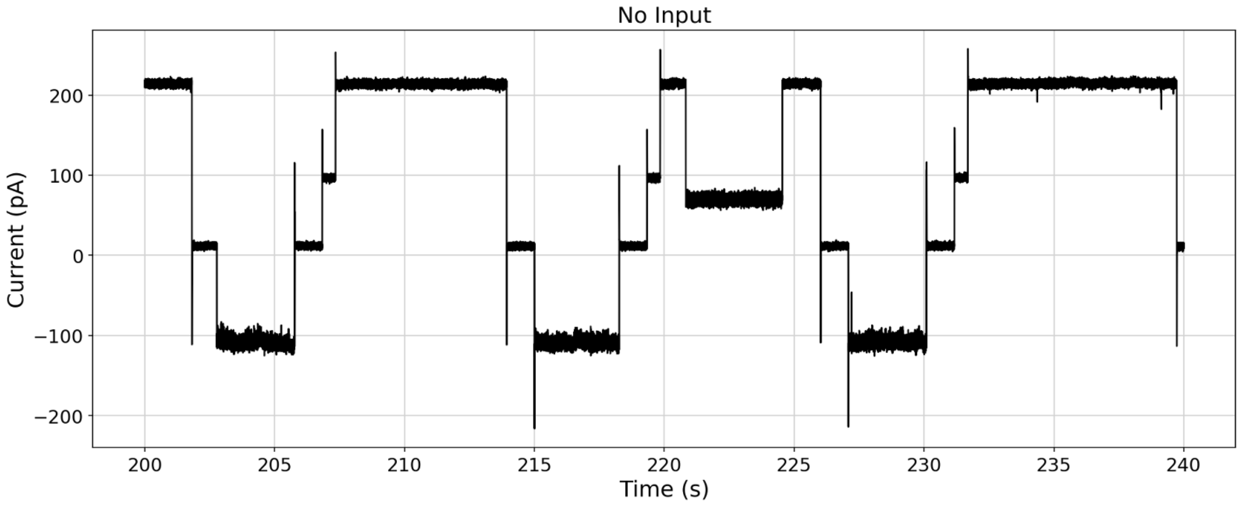

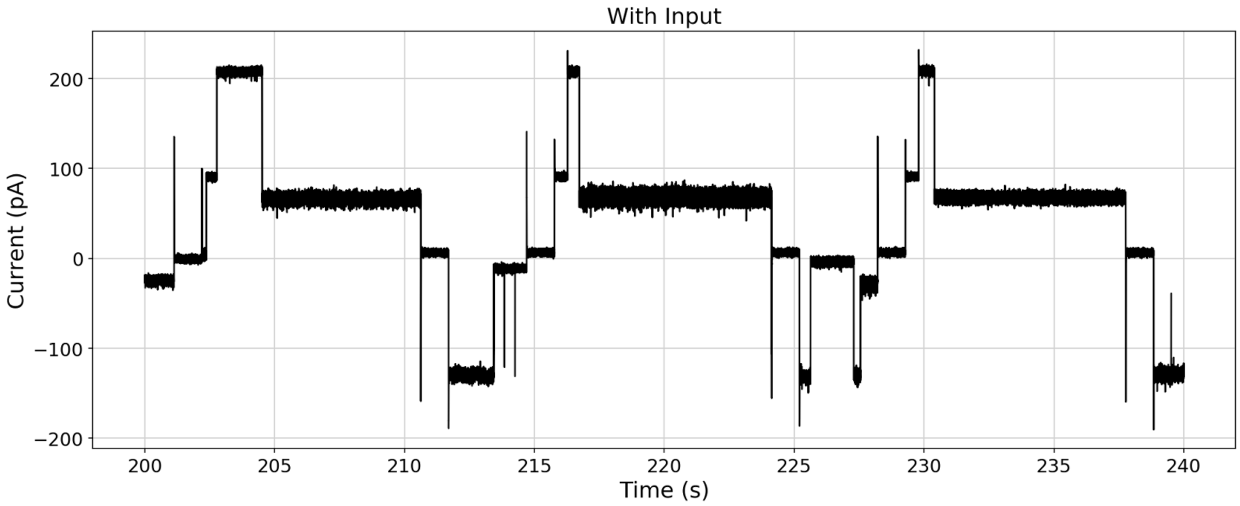

Supplementary Figure 2: Example nanopore raw traces from the single circuit kinetics experiment in Figure 2c. Upper plot is from the No Input sample and bottom plot is from the With Input sample. Both traces were collected at least 60 min into the run.

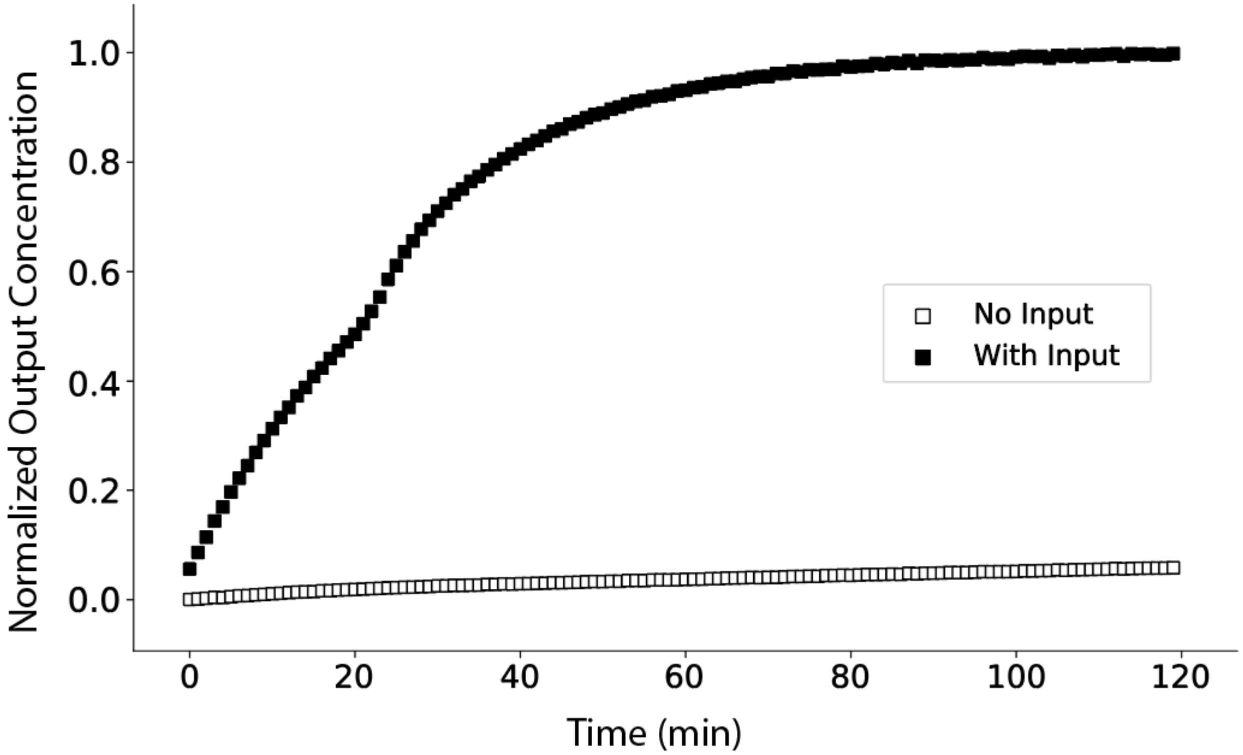

Supplementary Figure 3: Kinetics of a clamped catalytic DSD circuit measured on a fluorospectrometer. Addition of clamp domains mitigate circuit leakage caused by blunt end stacking.

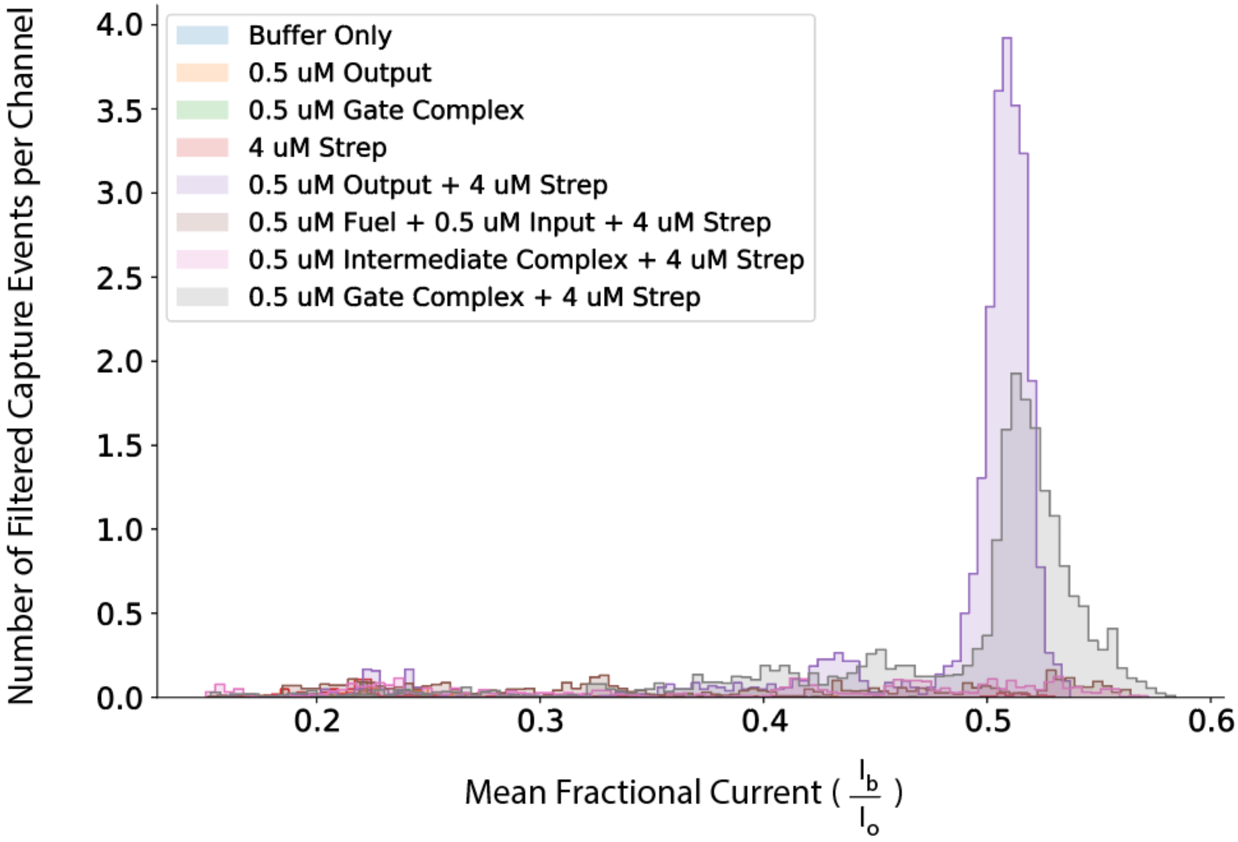

Supplementary Figure 4: Distribution of nanopore mean fractional current from filtered capture events. Each sample contains only the components described in the legend and were run in nanopore running buffer for 10 min each on a flow cell. All circuit components are from Circuit 4 with the output strand bearing Barcode C8.

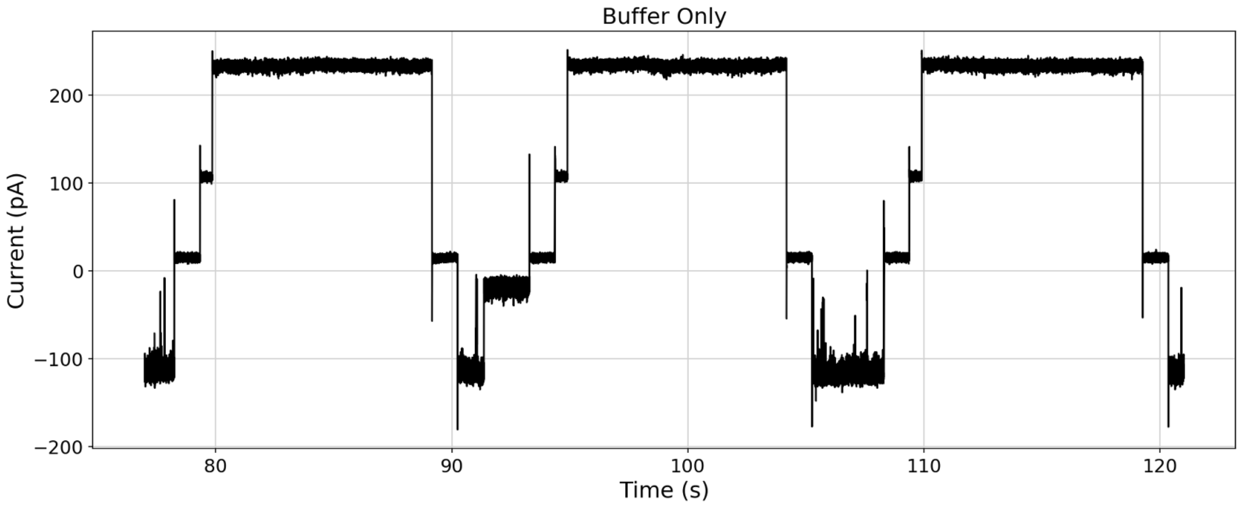

Buffer Only
Current (pA)
Time (s)

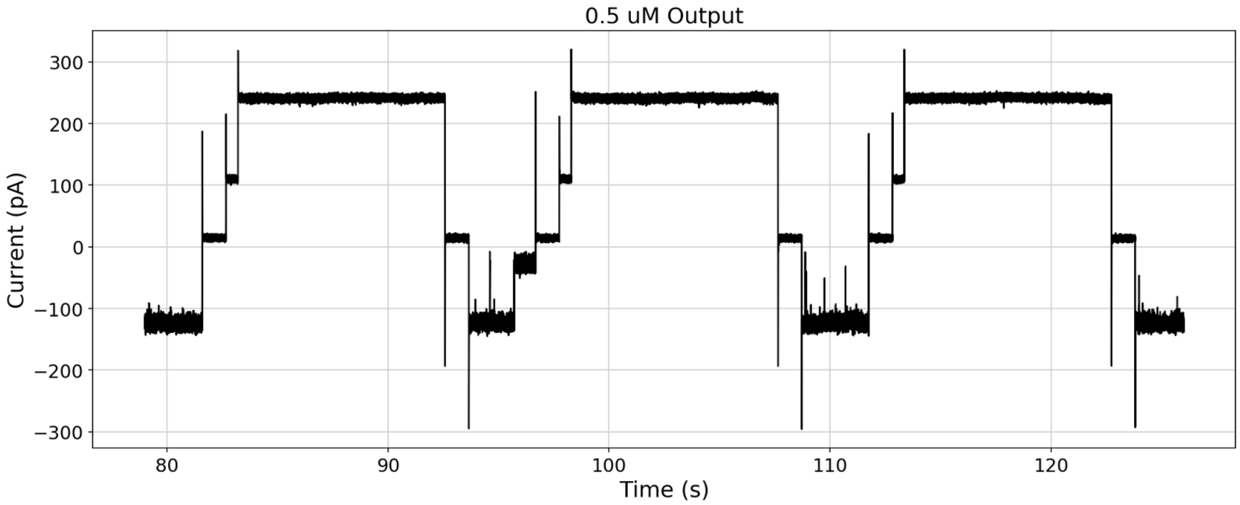

0.5 uM Output
Current (pA)
Time (s)

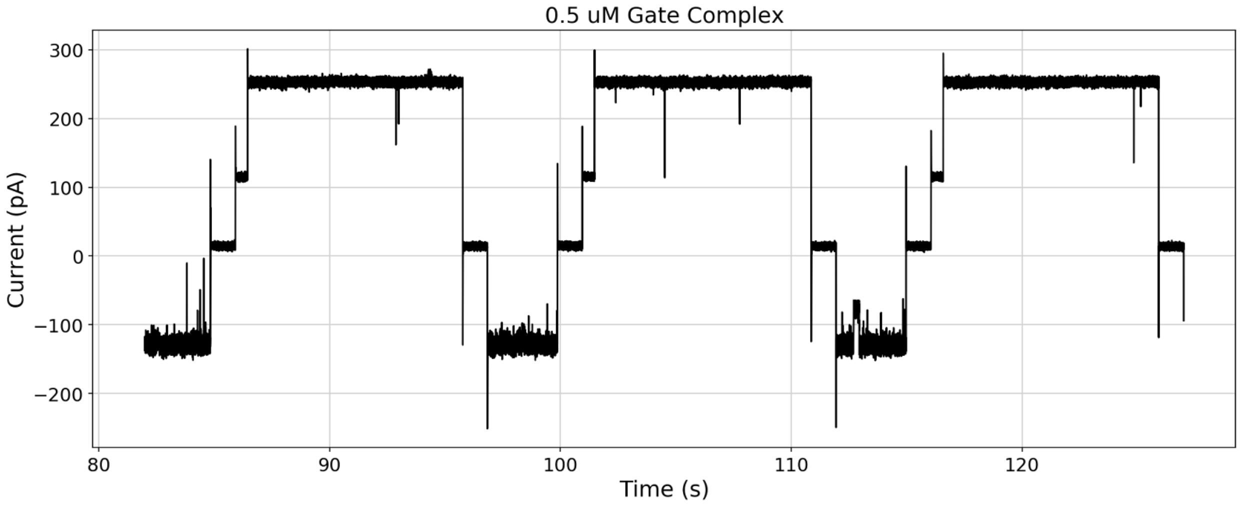

0.5 uM Gate Complex
Current (pA)
Time (s)

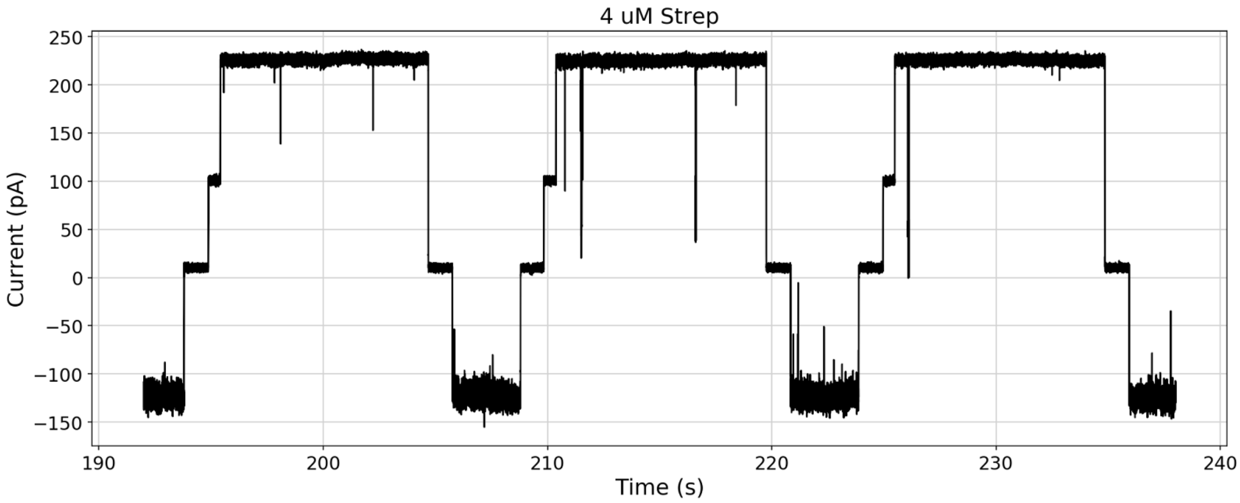
4 uM Strep
Current (pA)
Time (s)
250
200
150
100
50
0
−50
−100
−150
190
200
210
220
230
240

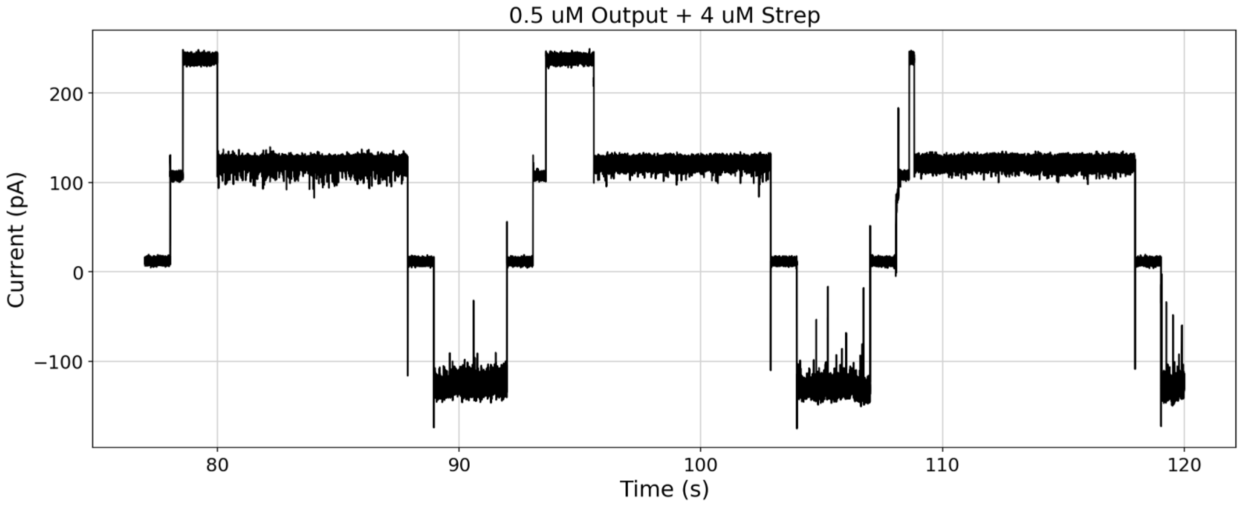
0.5 uM Output + 4 uM Strep
Current (pA)
Time (s)
200
100
0
−100
80
90
100
110
120

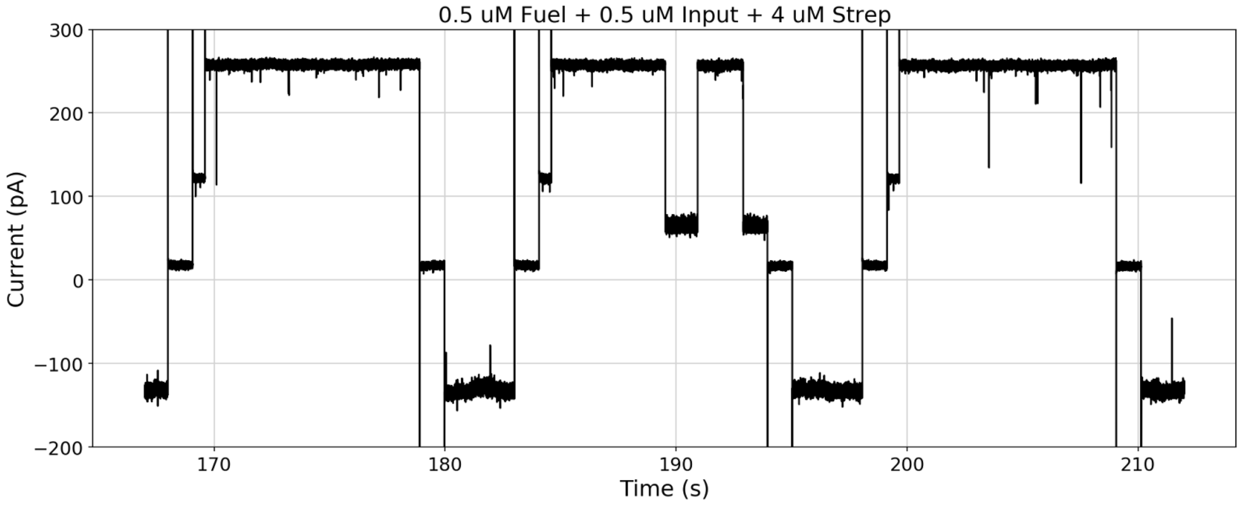
0.5 uM Fuel + 0.5 uM Input + 4 uM Strep
Current (pA)
Time (s)
300
200
100
0
−100
−200
170
180
190
200
210

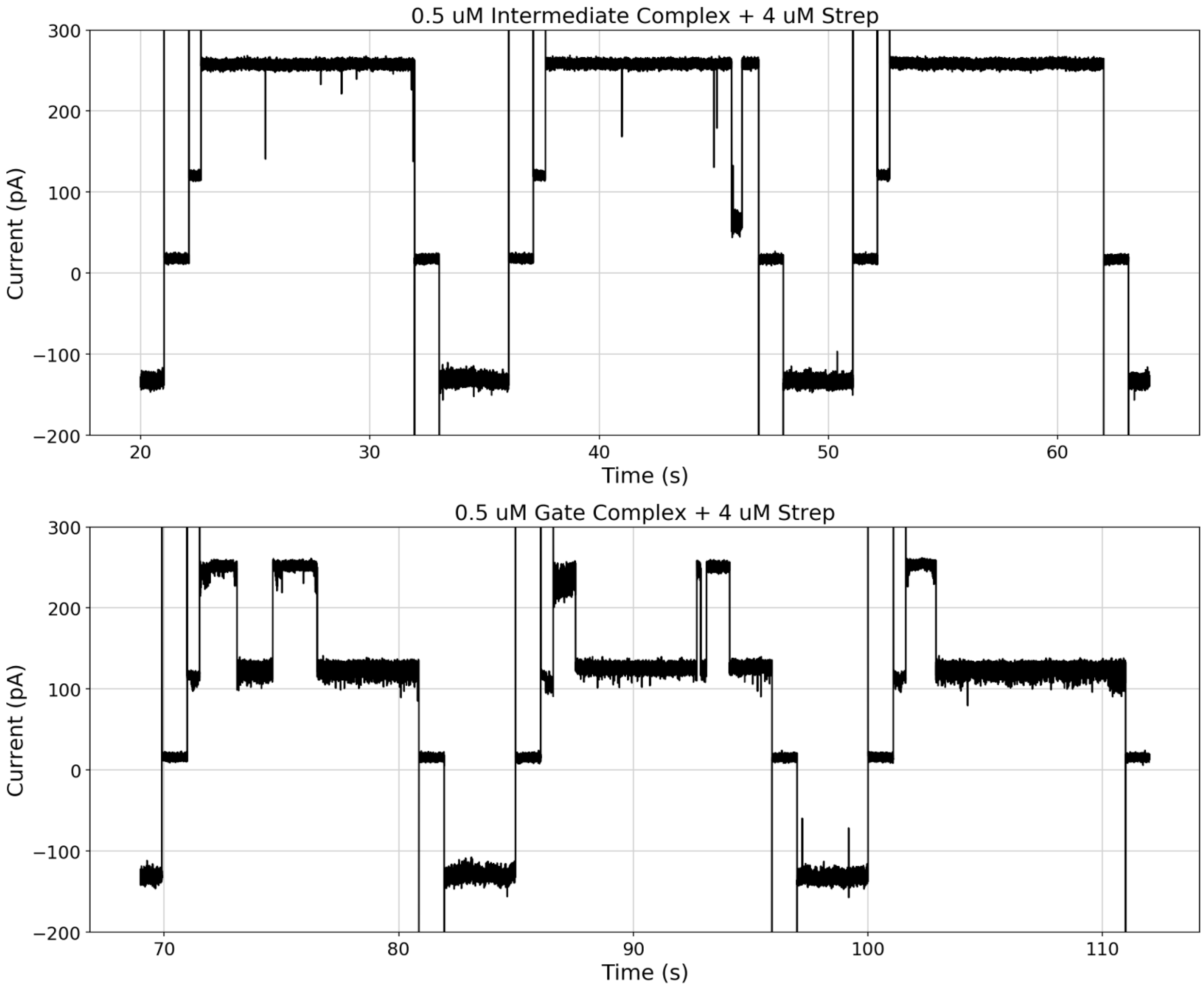

Supplementary Figure 5: Example nanopore raw traces for each sample in Supplementary Figure 4 where circuit components were run independently on the nanopore for 10 min each.

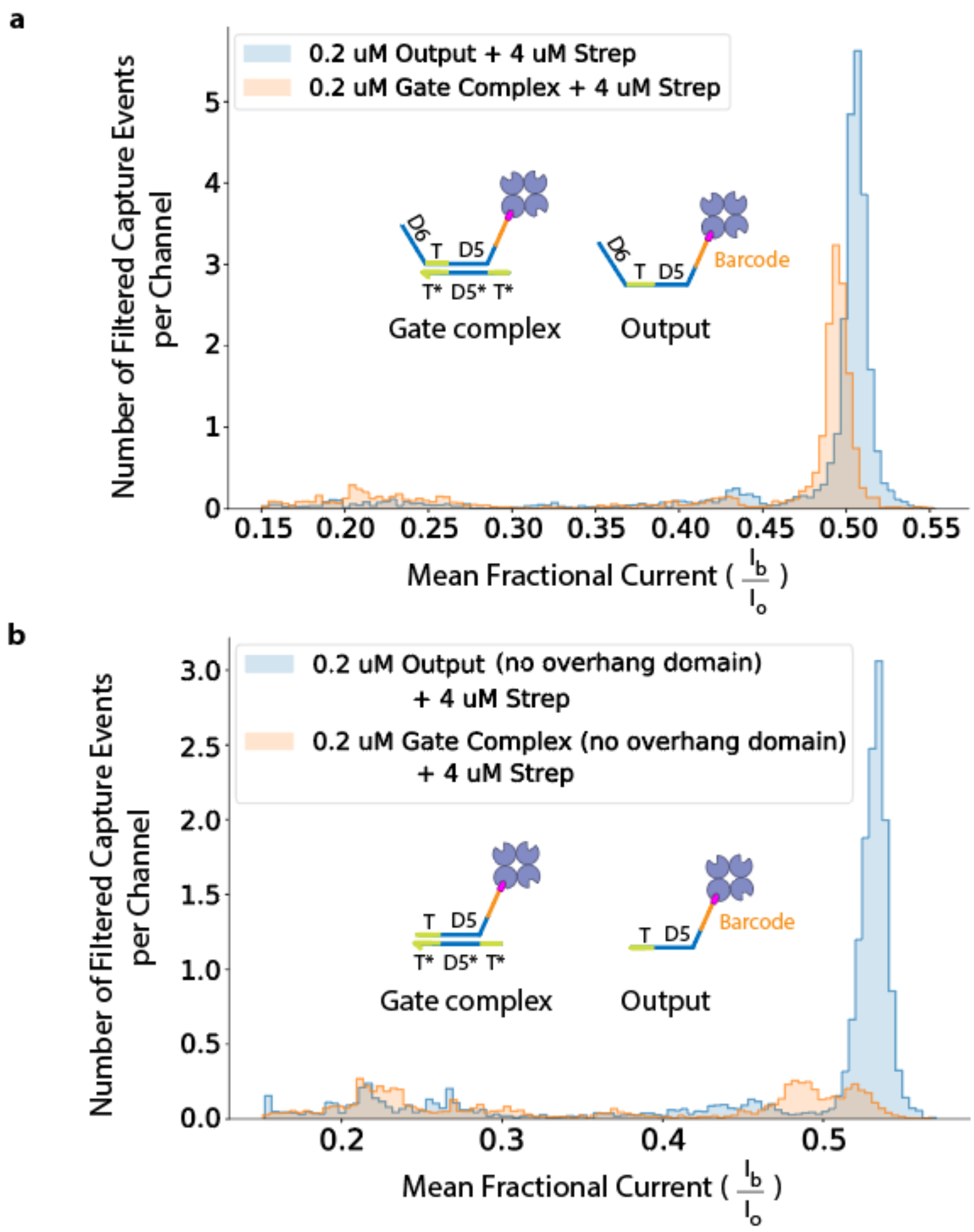

Supplementary Figure 6: Distribution of nanopore mean fractional current from filtered capture events for two sets of samples, each comparing free output strand captures to gate complex captures. **a)** The first set compares 0.2 uM of free output strand to 0.2 uM of gate complex. **b)** The second set compares 0.2 uM of free output strand to 0.2 uM gate complex without the D6 overhang domain. All samples contained 4 uM streptavidin and were run on the flow cell for 10 min. All circuit components are from Circuit 4 with the output strand bearing Barcode C8.

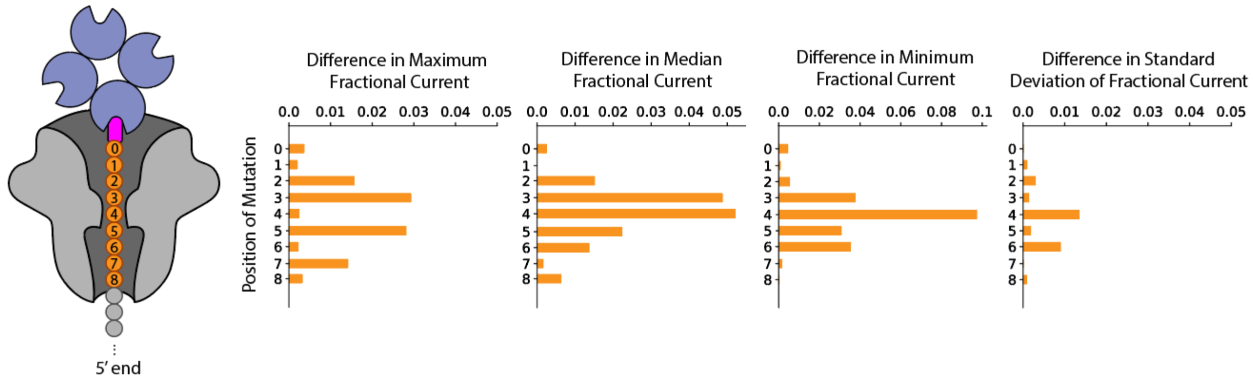

Supplementary Figure 7: Each plot depicts the change in either maximum, median, minimum, or standard deviation of nanopore fractional current elicited by a single-nucleotide mutation at each position on the strand's barcode.

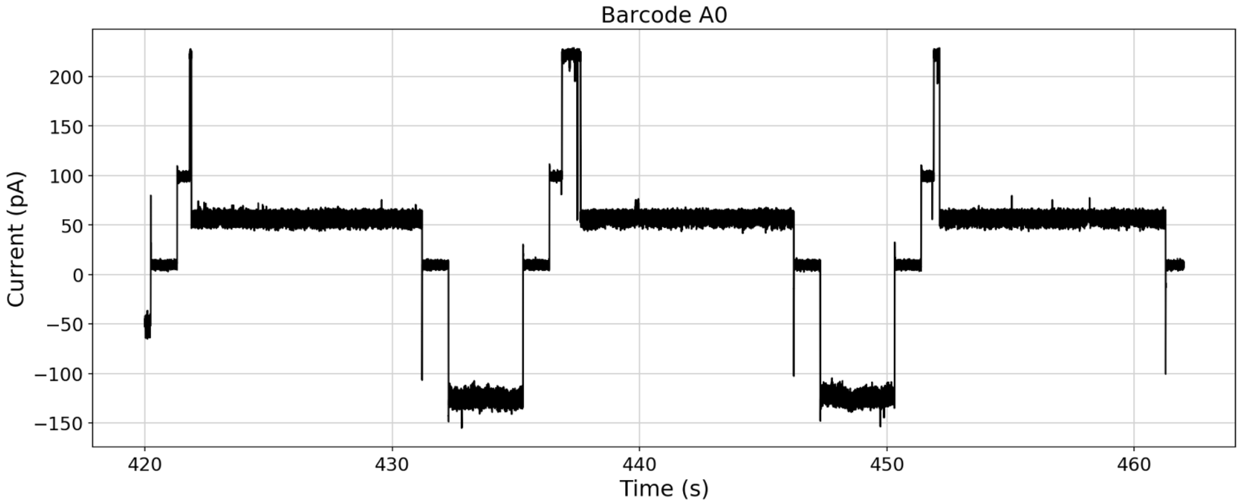
Barcode A0
Current (pA)
200
150
100
50
0
−50
−100
−150
420
430
440
450
460
Time (s)

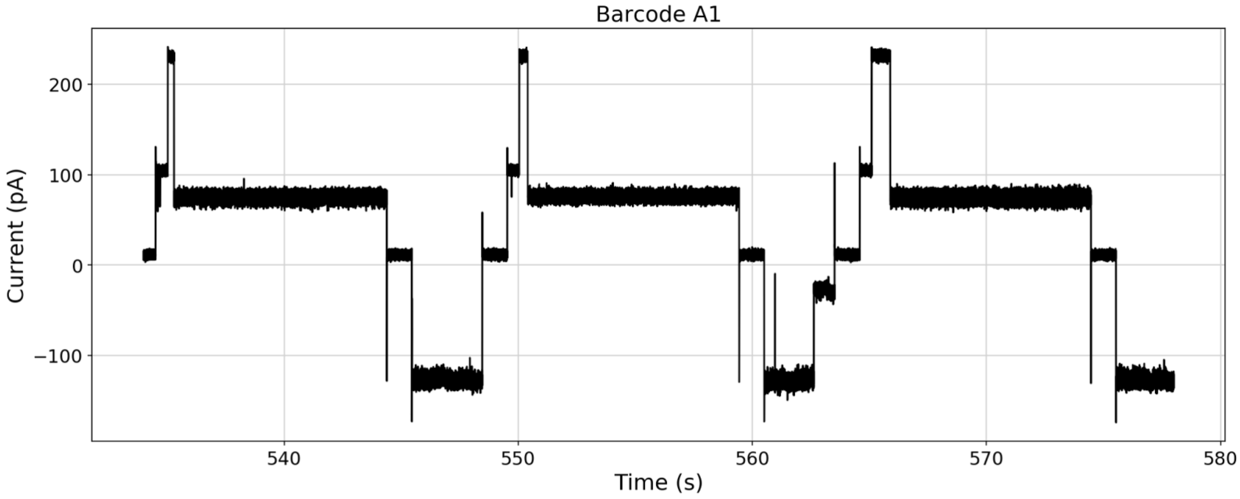
Barcode A1
Current (pA)
200
100
0
−100
540
550
560
570
580
Time (s)

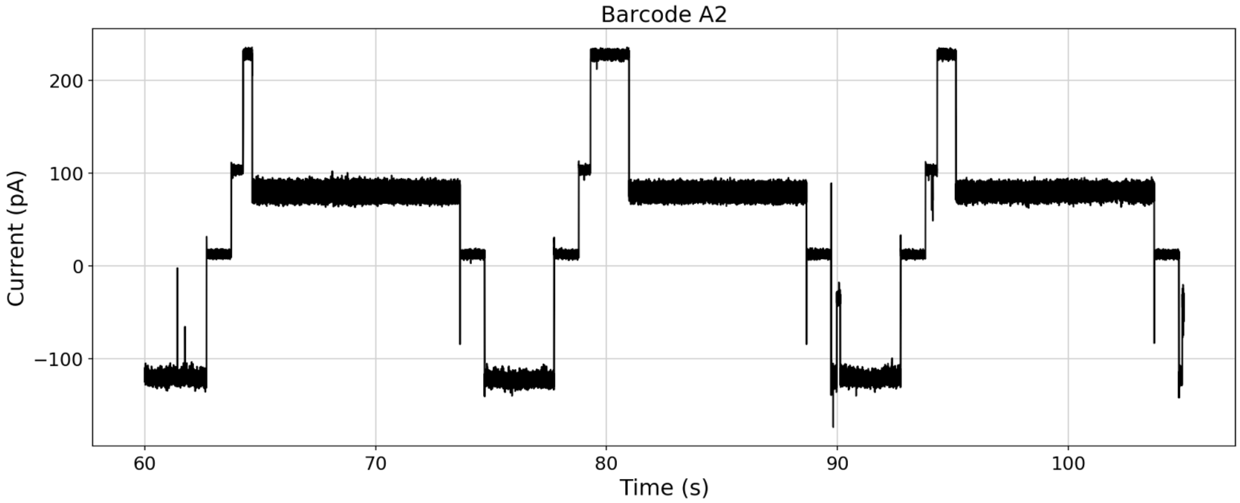
Barcode A2
Current (pA)
200
100
0
−100
60
70
80
90
100
Time (s)

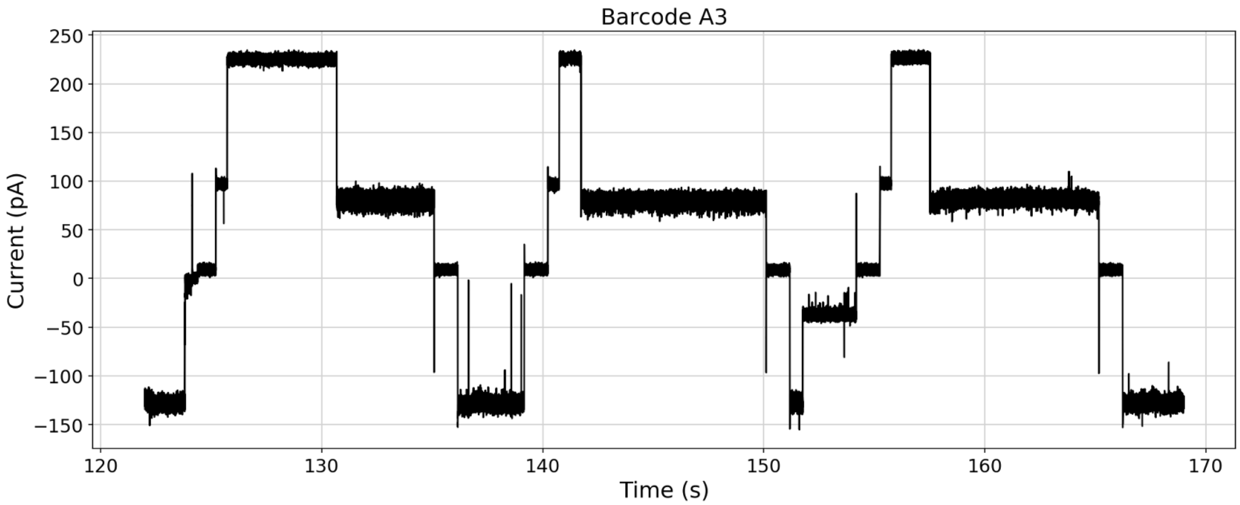
Barcode A3
Current (pA)
Time (s)

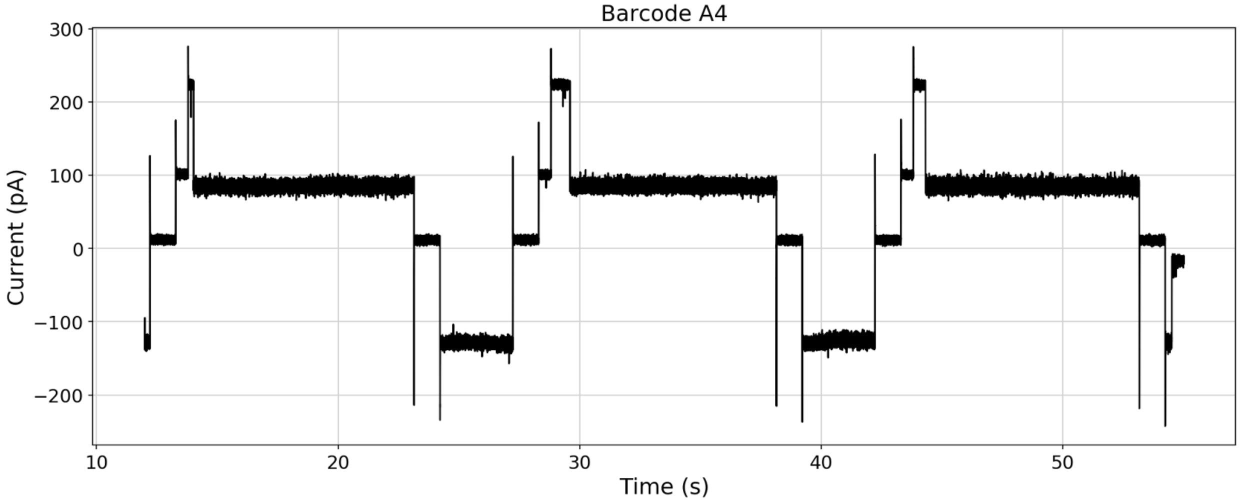
Barcode A4
Current (pA)
Time (s)

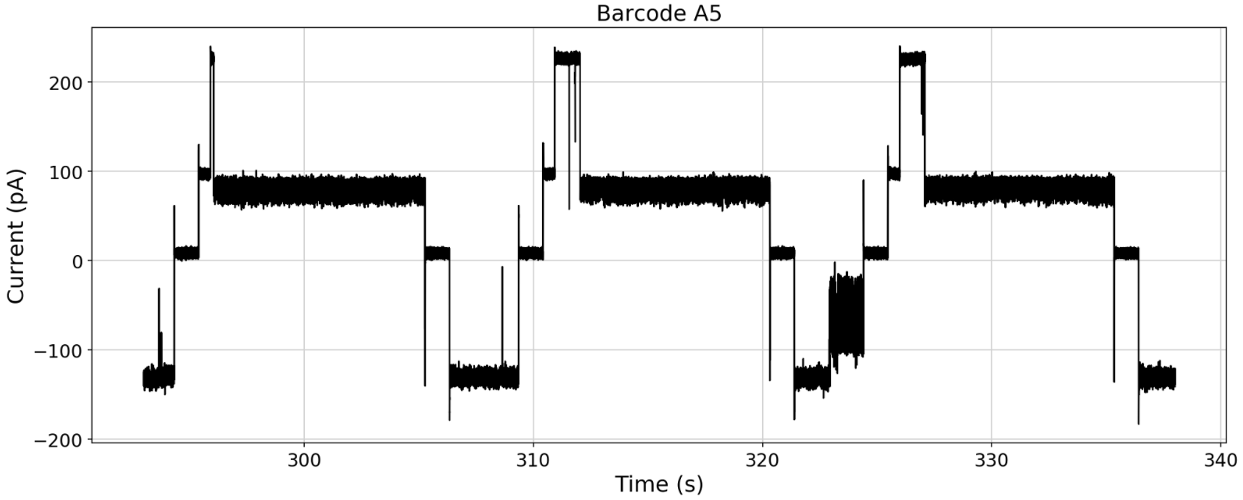
Barcode A5
Current (pA)
Time (s)

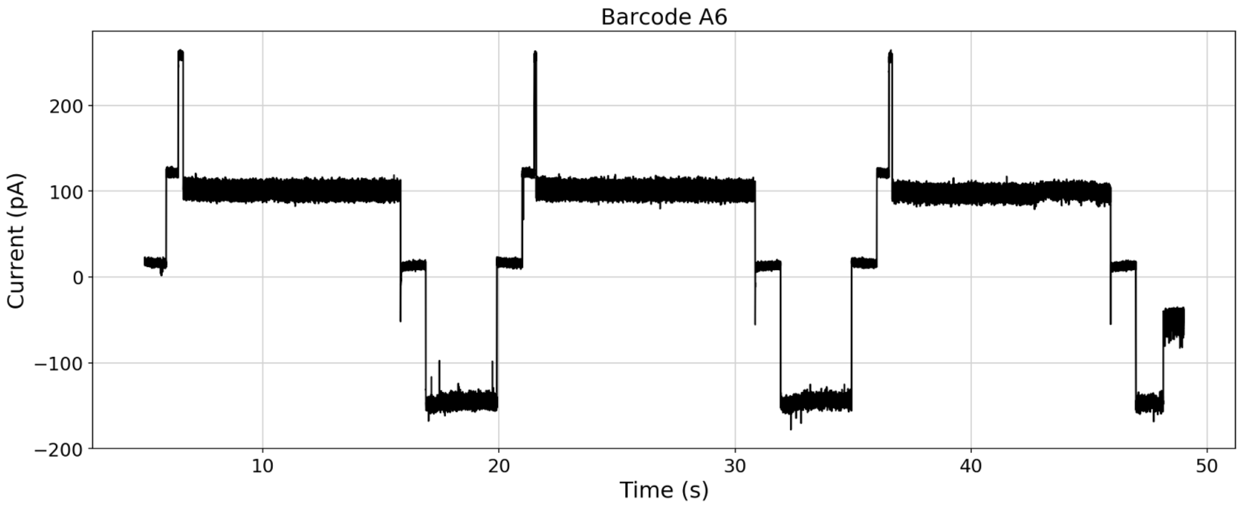
Barcode A6
Current (pA)
Time (s)

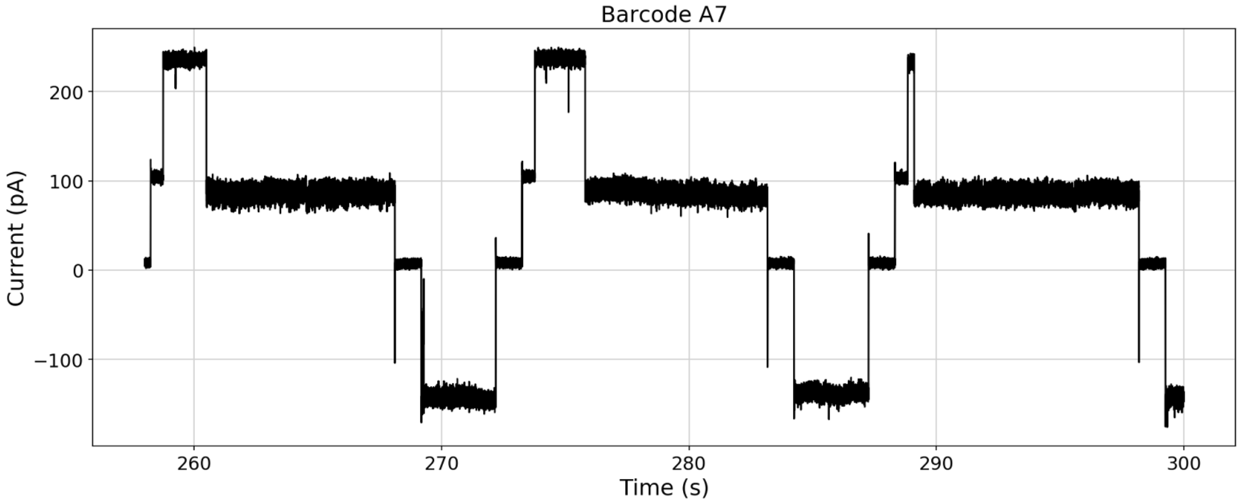
Barcode A7
Current (pA)
Time (s)

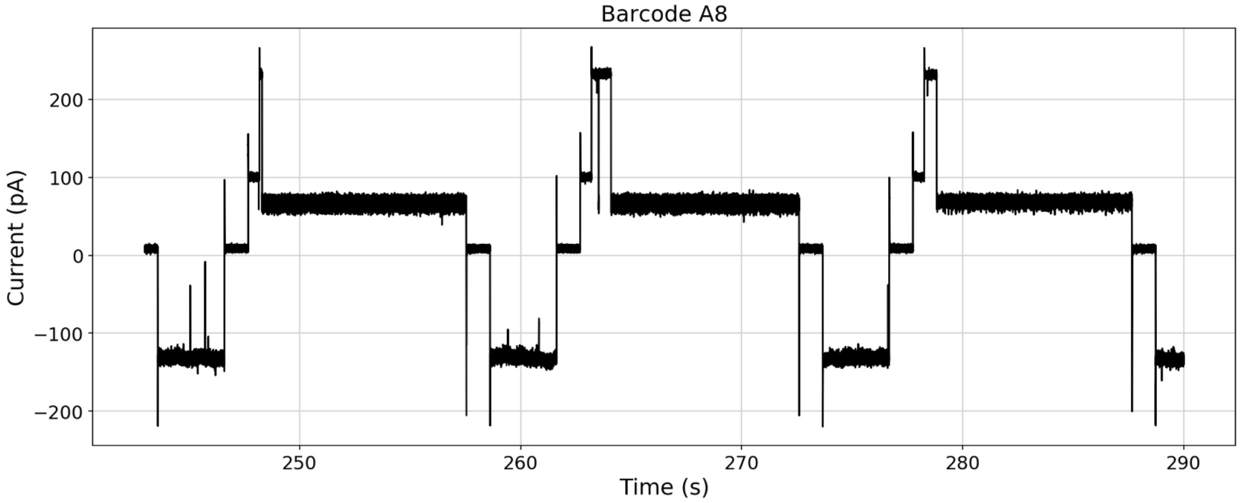
Barcode A8
Current (pA)
Time (s)

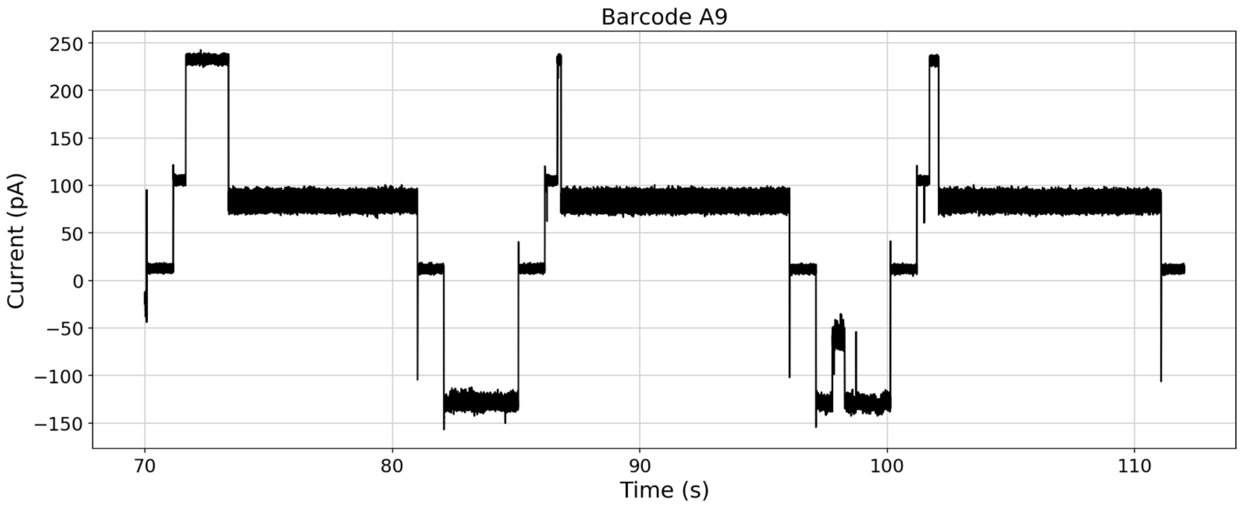
Barcode A9
Current (pA)
250
200
150
100
50
0
−50
−100
−150
70
80
90
100
110
Time (s)

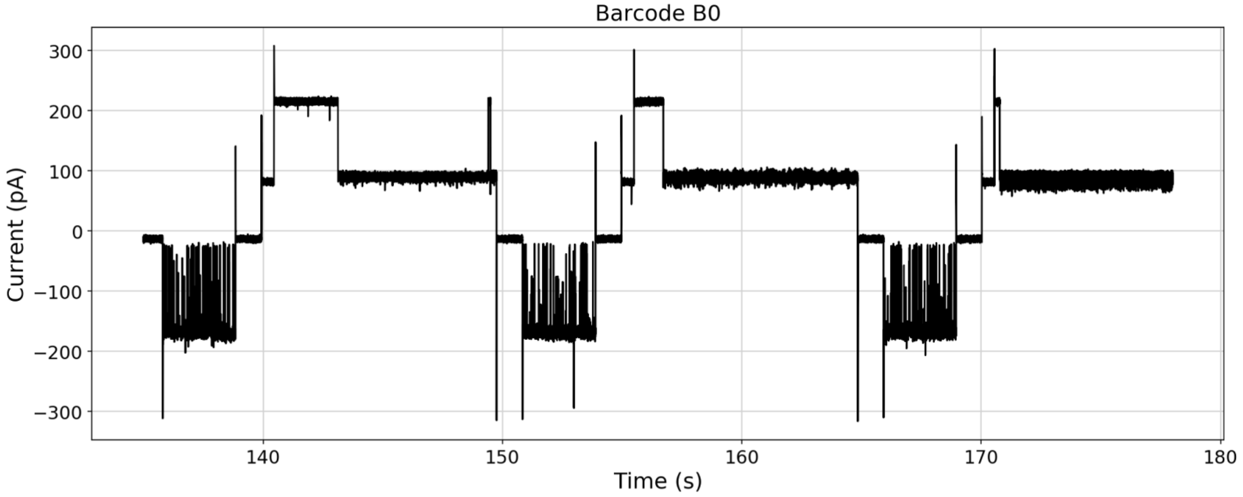
Barcode B0
Current (pA)
300
200
100
0
−100
−200
−300
140
150
160
170
180
Time (s)

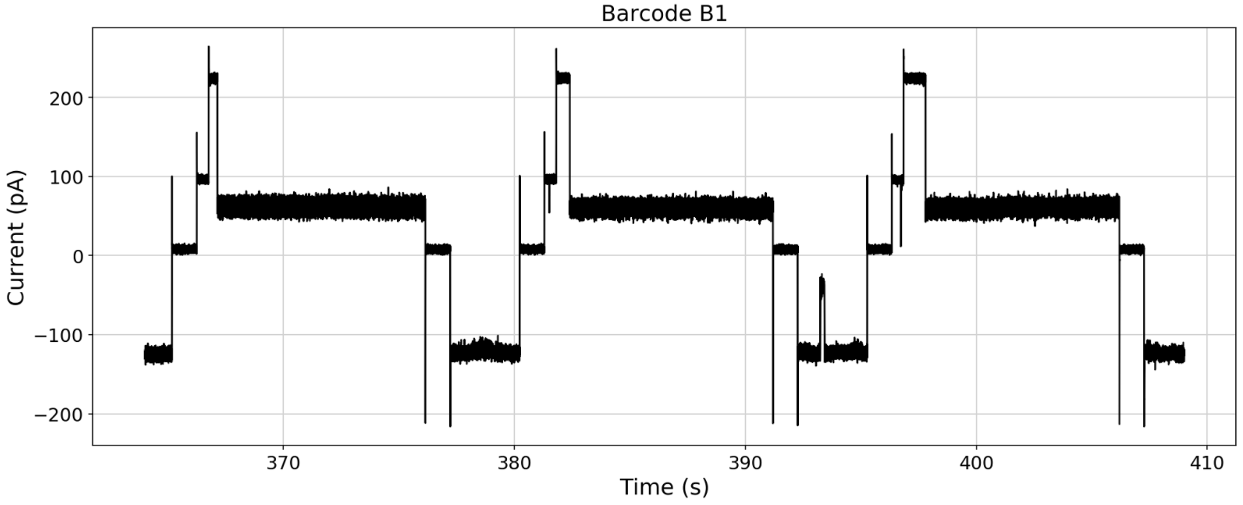
Barcode B1
Current (pA)
200
100
0
−100
−200
370
380
390
400
410
Time (s)

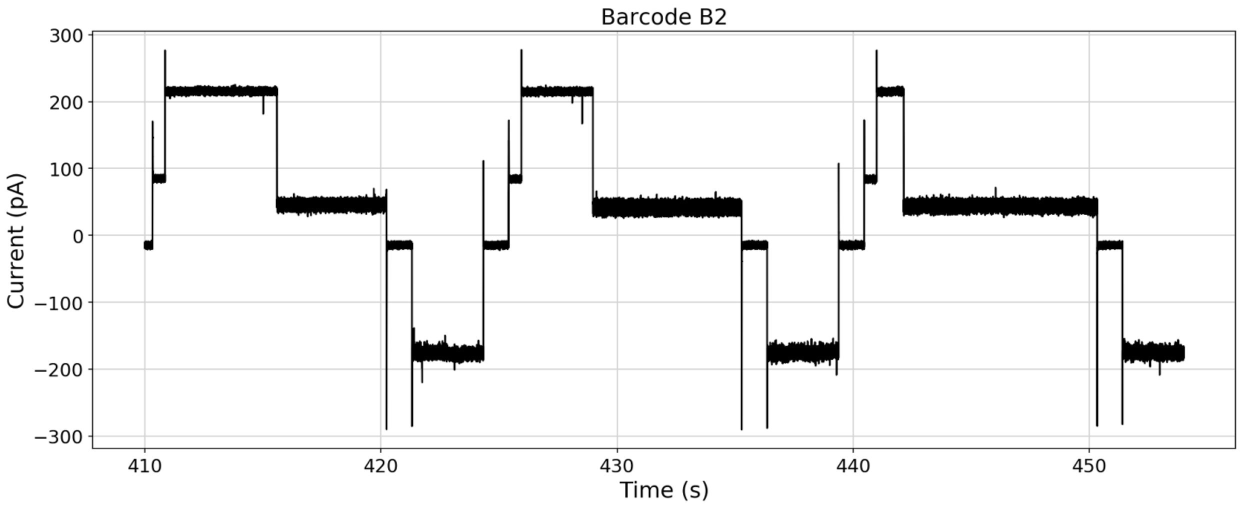
Barcode B2
Current (pA)
Time (s)

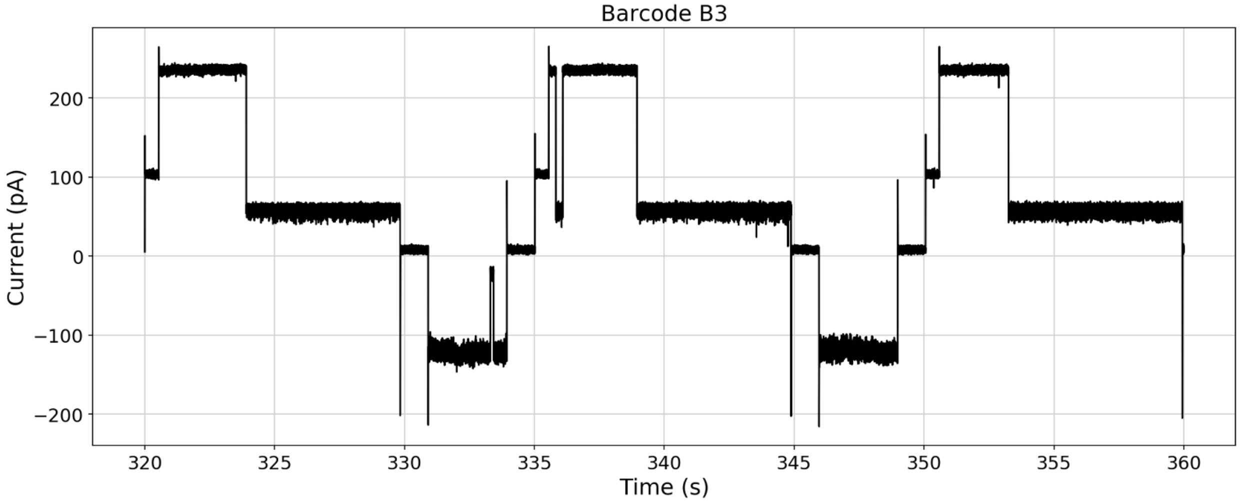
Barcode B3
Current (pA)
Time (s)

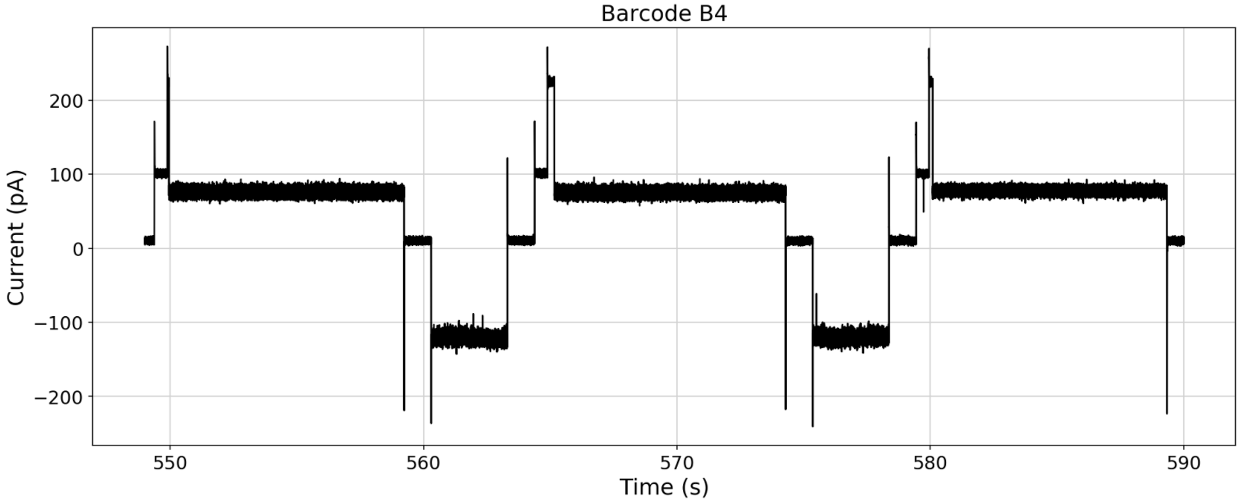
Barcode B4
Current (pA)
Time (s)

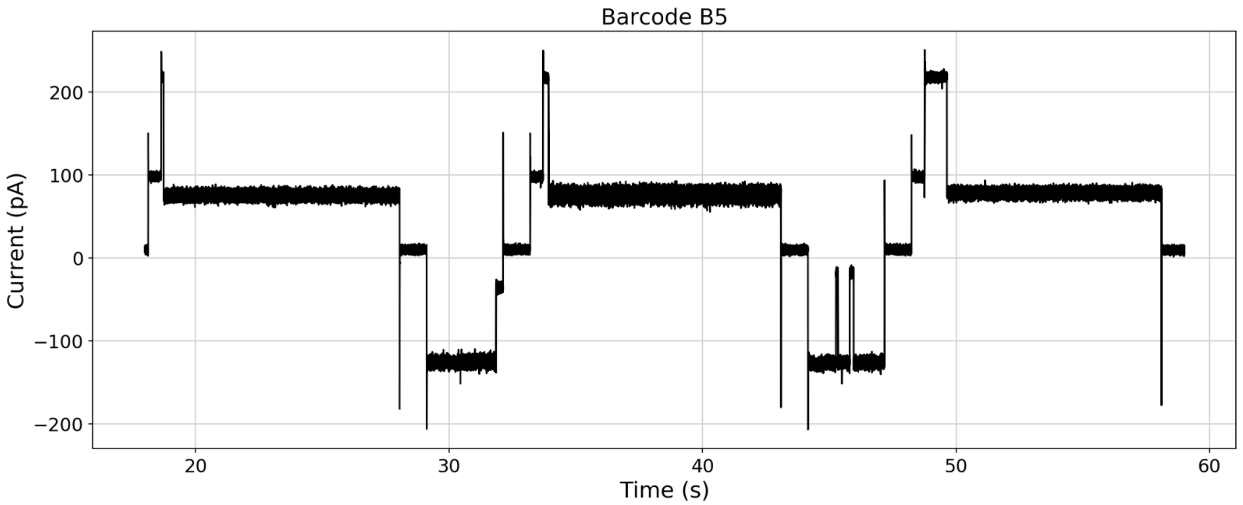
Barcode B5
Current (pA)
200
100
0
−100
−200
20
30
40
50
60
Time (s)

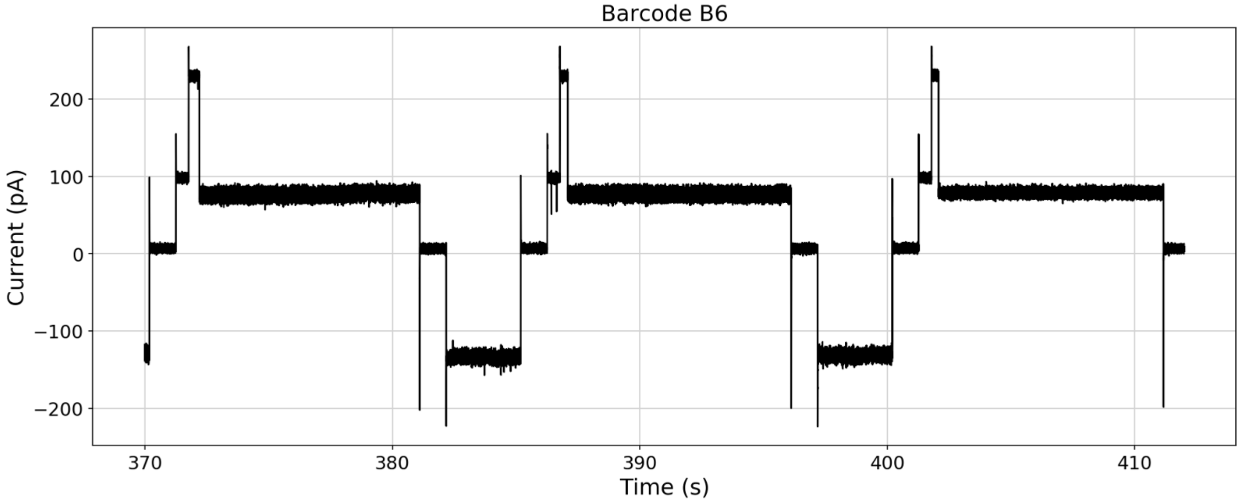
Barcode B6
Current (pA)
200
100
0
−100
−200
370
380
390
400
410
Time (s)

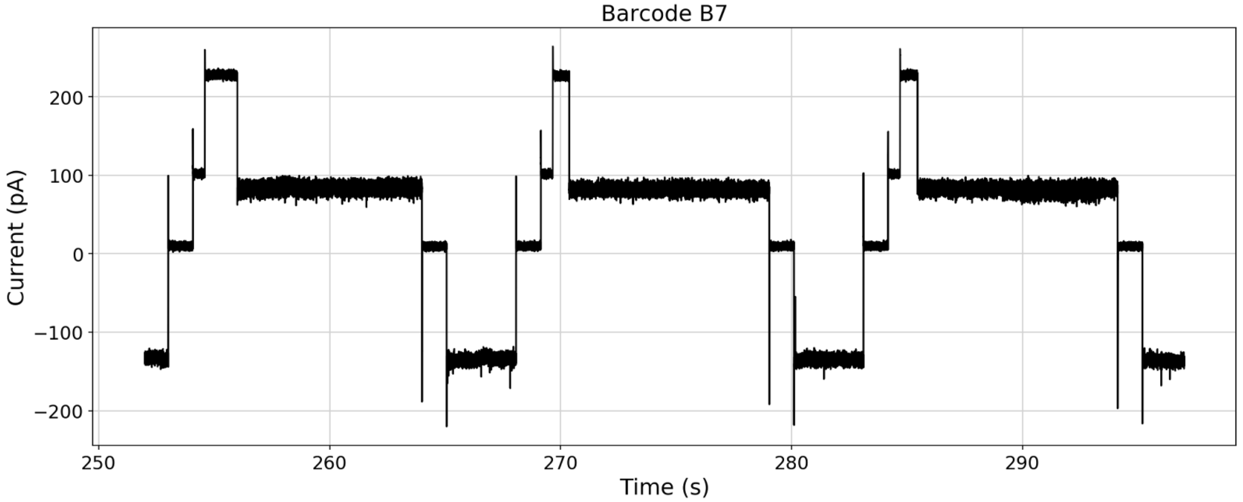
Barcode B7
Current (pA)
200
100
0
−100
−200
250
260
270
280
290
Time (s)

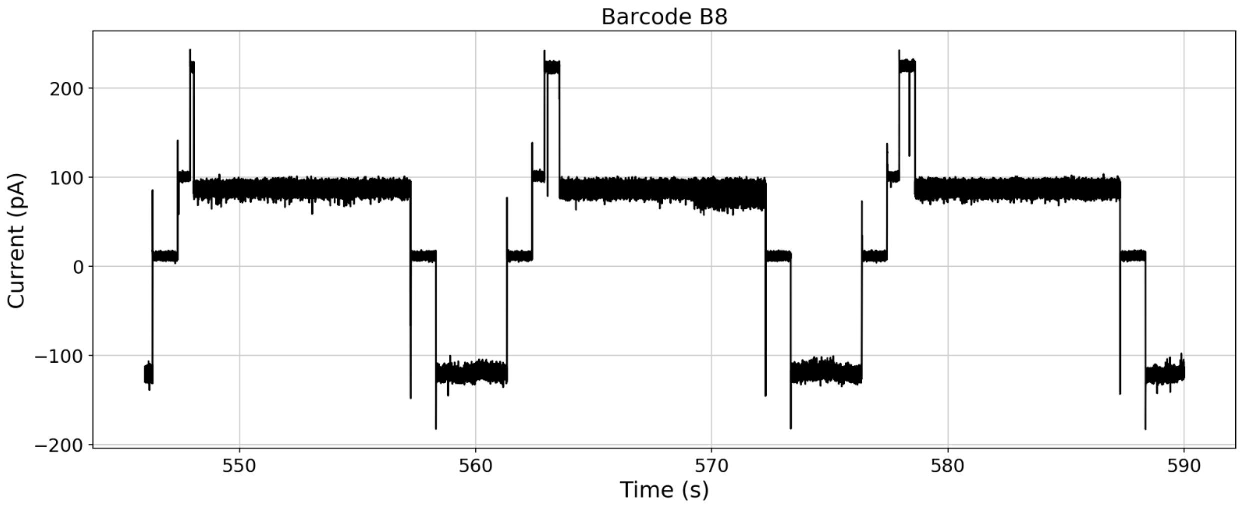
Barcode B8
Current (pA)
Time (s)
200
100
0
−100
−200
550
560
570
580
590

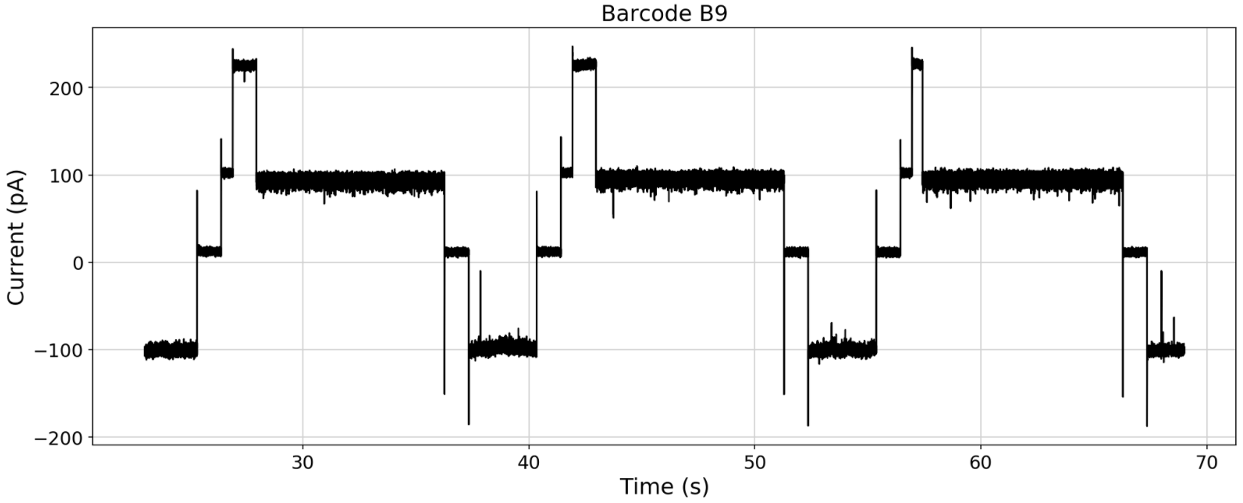
Barcode B9
Current (pA)
Time (s)
200
100
0
−100
−200
30
40
50
60
70

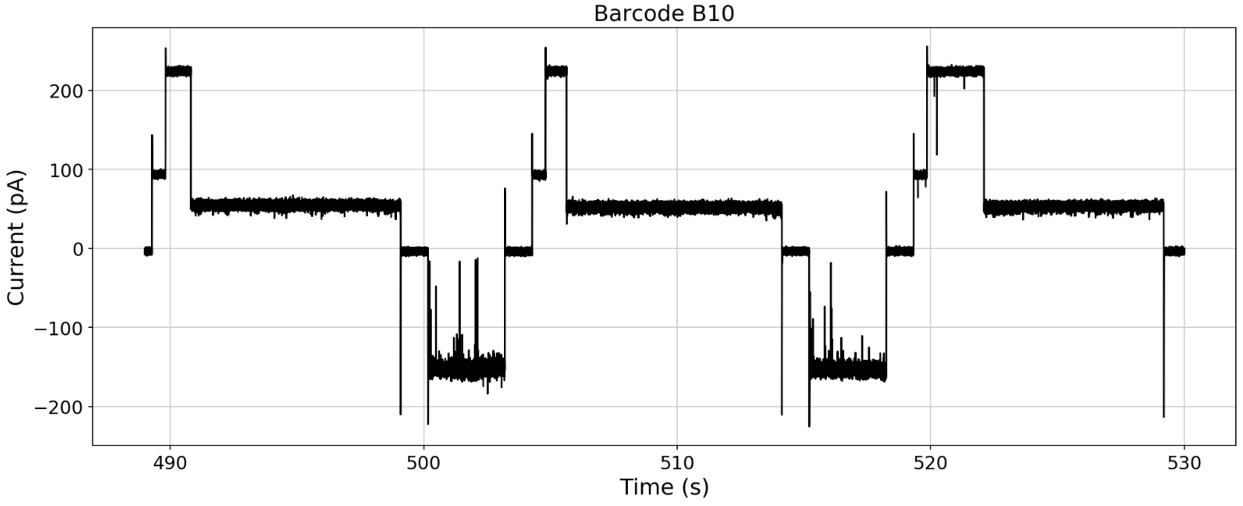
Barcode B10
Current (pA)
Time (s)
200
100
0
−100
−200
490
500
510
520
530

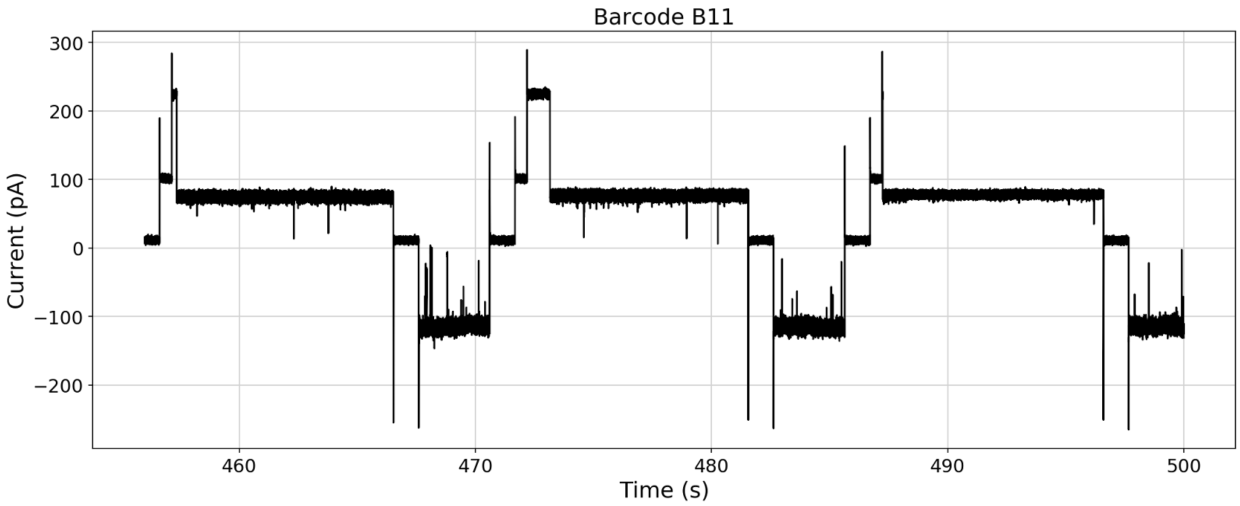
Barcode B11
Current (pA)
Time (s)

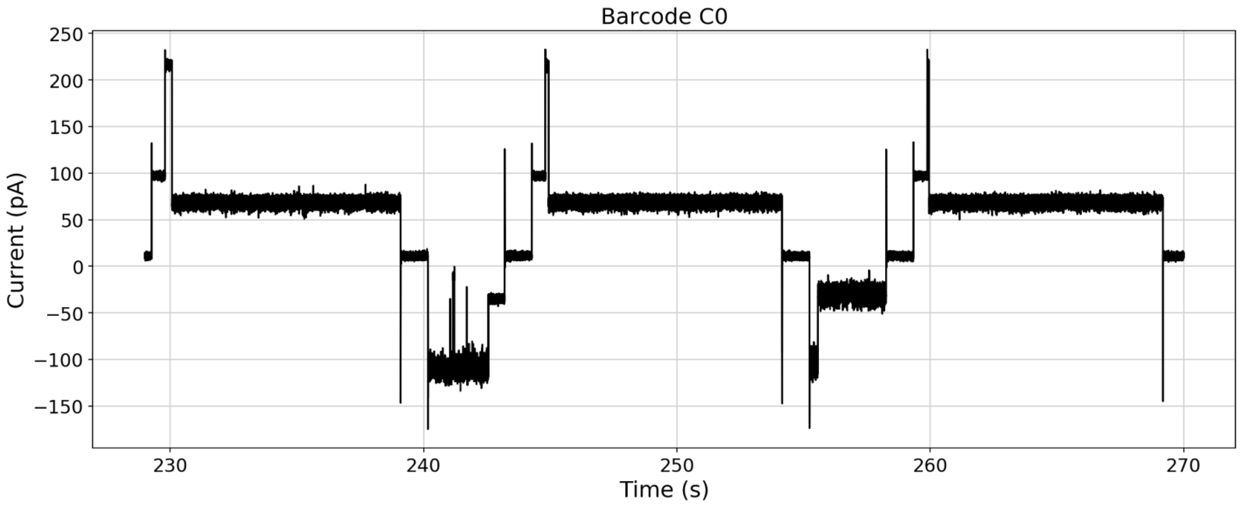
Barcode C0
Current (pA)
Time (s)

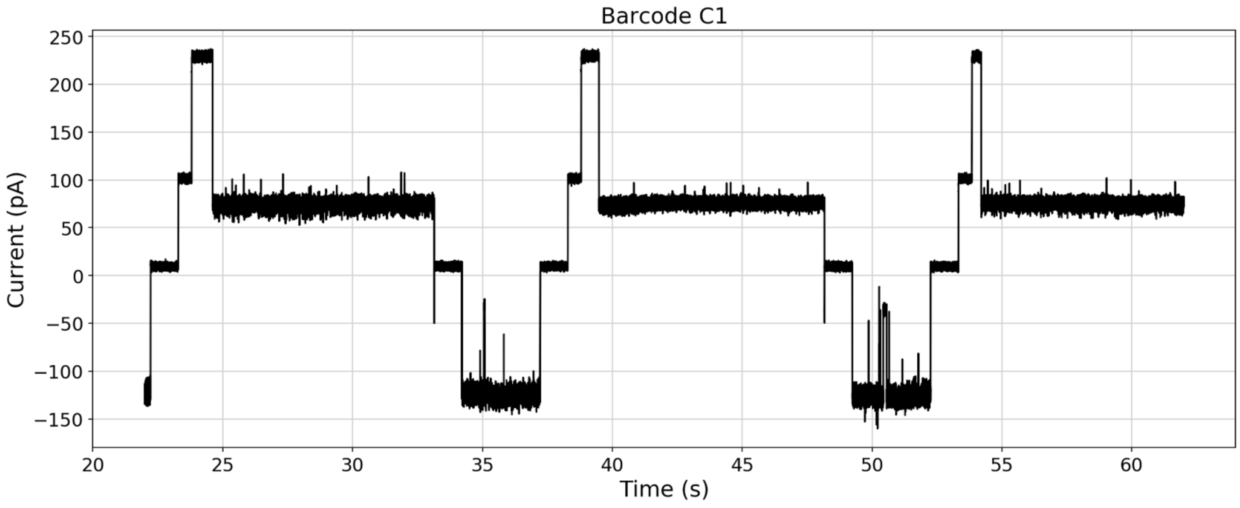
Barcode C1
Current (pA)
Time (s)

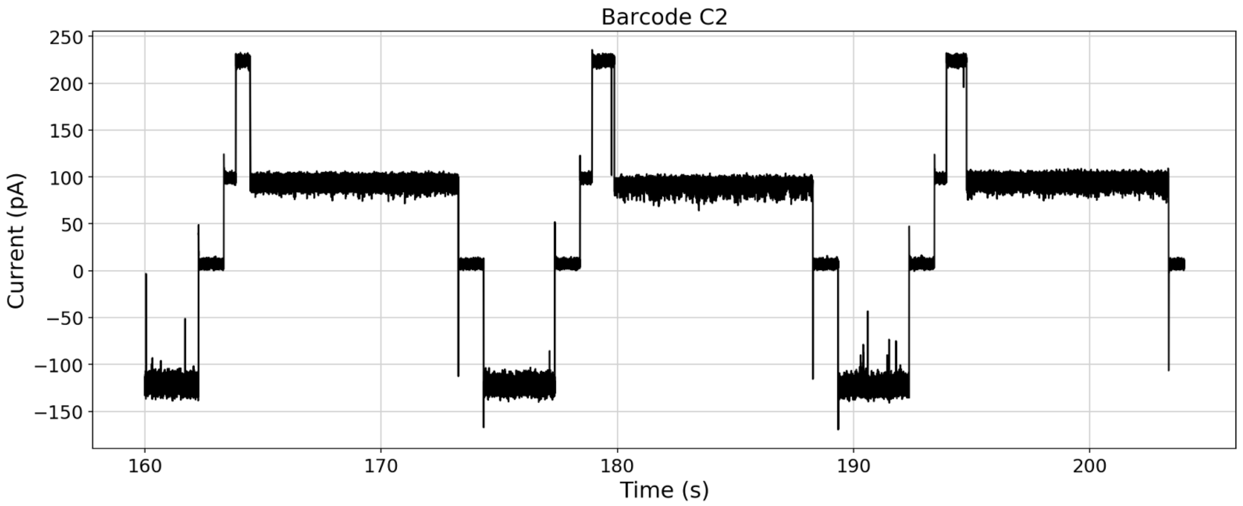
Barcode C2
Current (pA)
Time (s)
250
200
150
100
50
0
−50
−100
−150
160
170
180
190
200

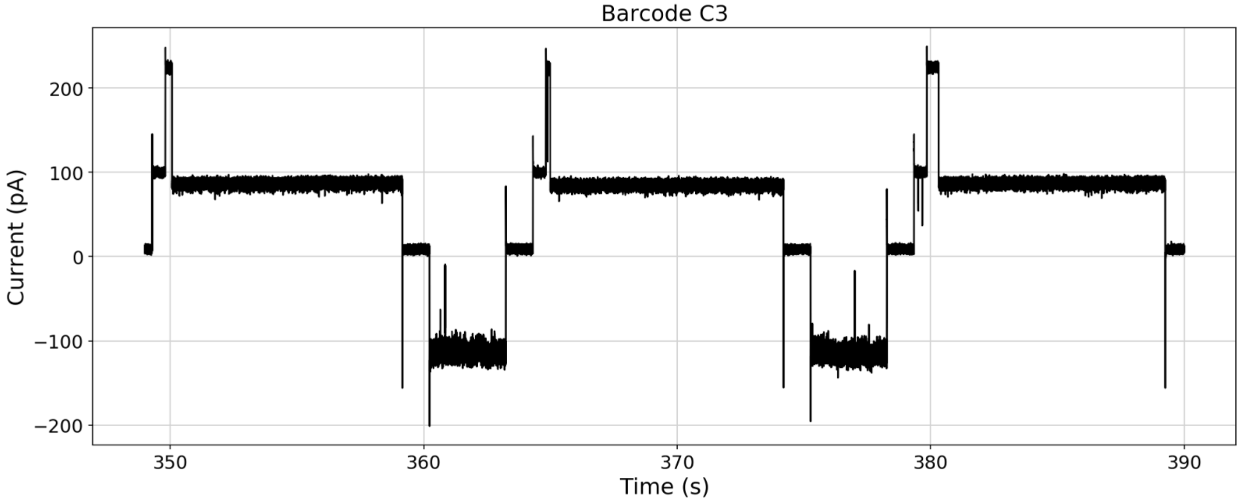
Barcode C3
Current (pA)
Time (s)
200
100
0
−100
−200
350
360
370
380
390

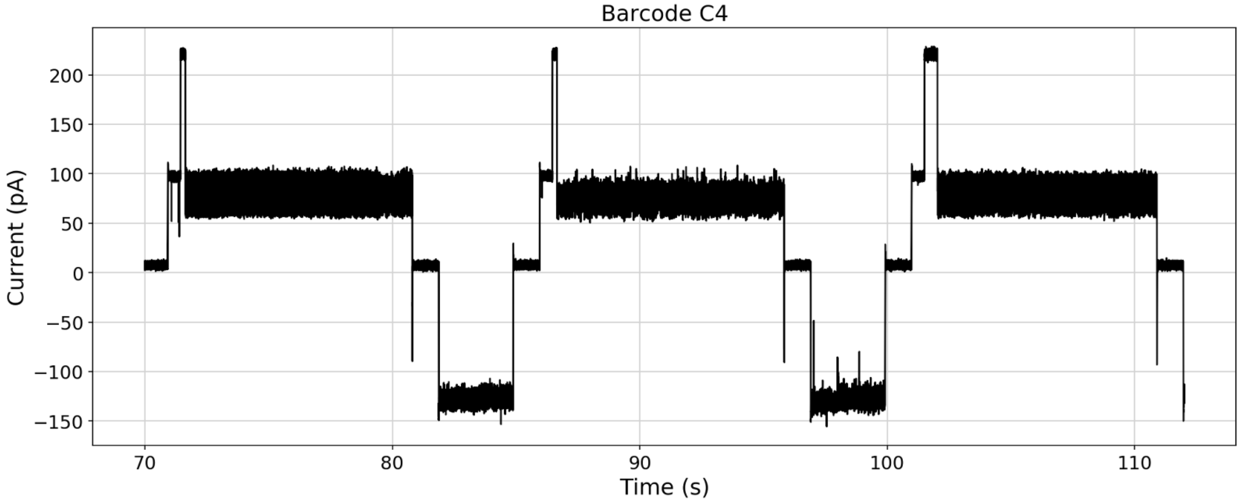
Barcode C4
Current (pA)
Time (s)
200
150
100
50
0
−50
−100
−150
70
80
90
100
110

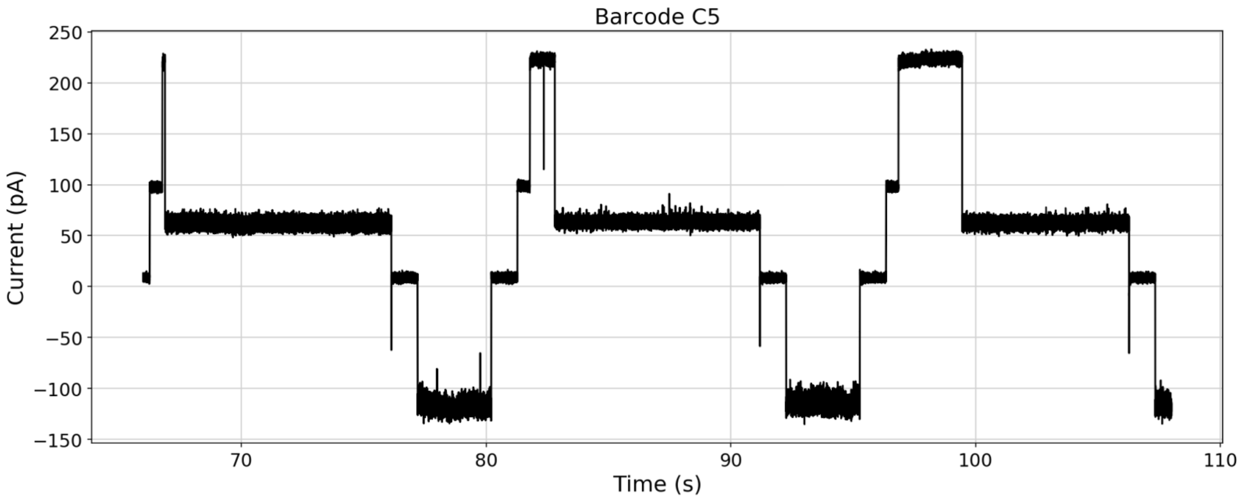
Barcode C5
Current (pA)
Time (s)
250
200
150
100
50
0
−50
−100
−150
70
80
90
100
110

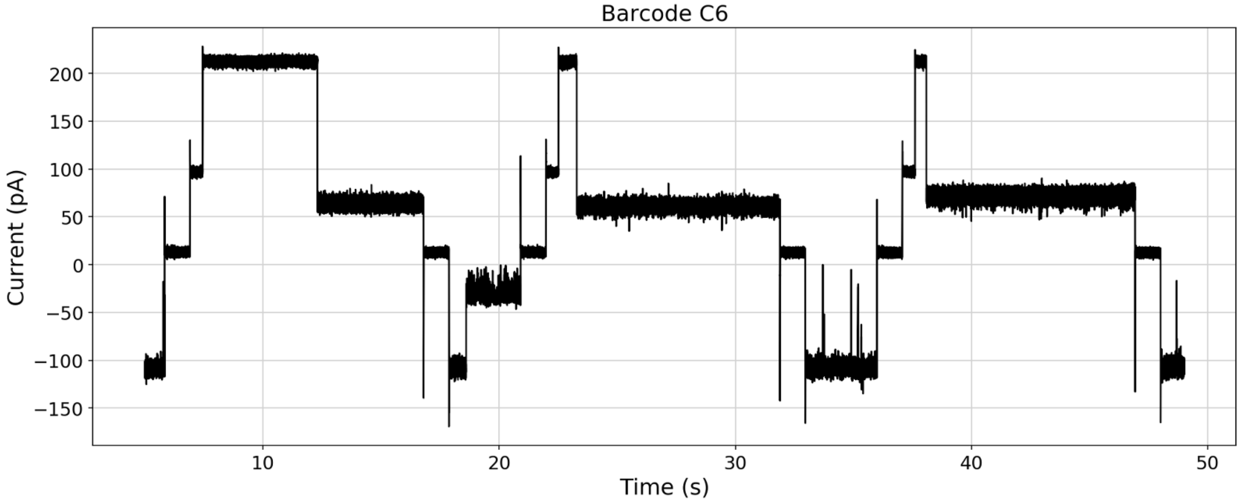
Barcode C6
Current (pA)
Time (s)
200
150
100
50
0
−50
−100
−150
10
20
30
40
50

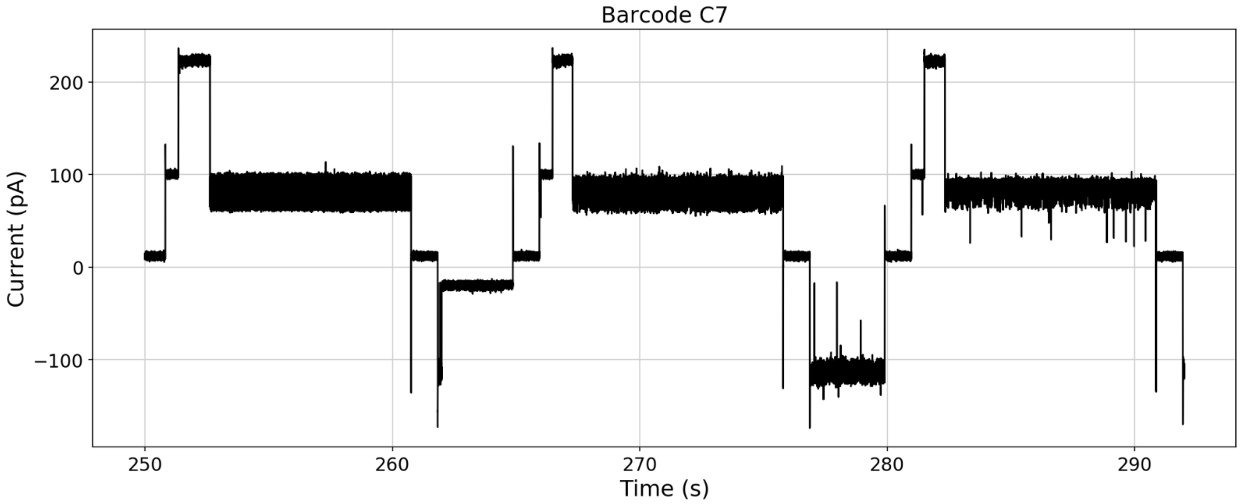
Barcode C7
Current (pA)
Time (s)
200
100
0
−100
250
260
270
280
290

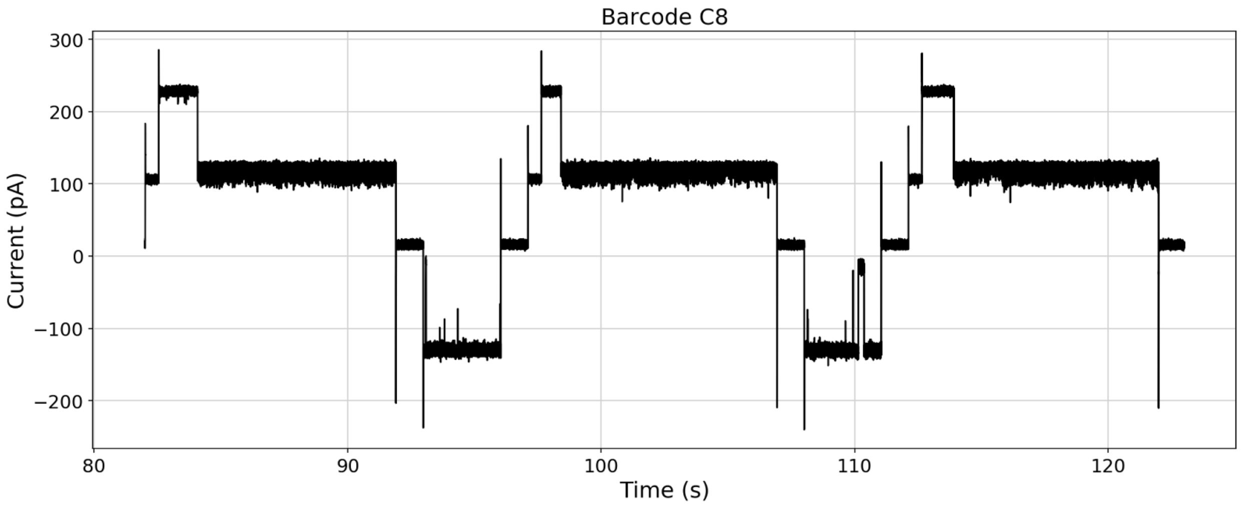
Barcode C8
Current (pA)
Time (s)

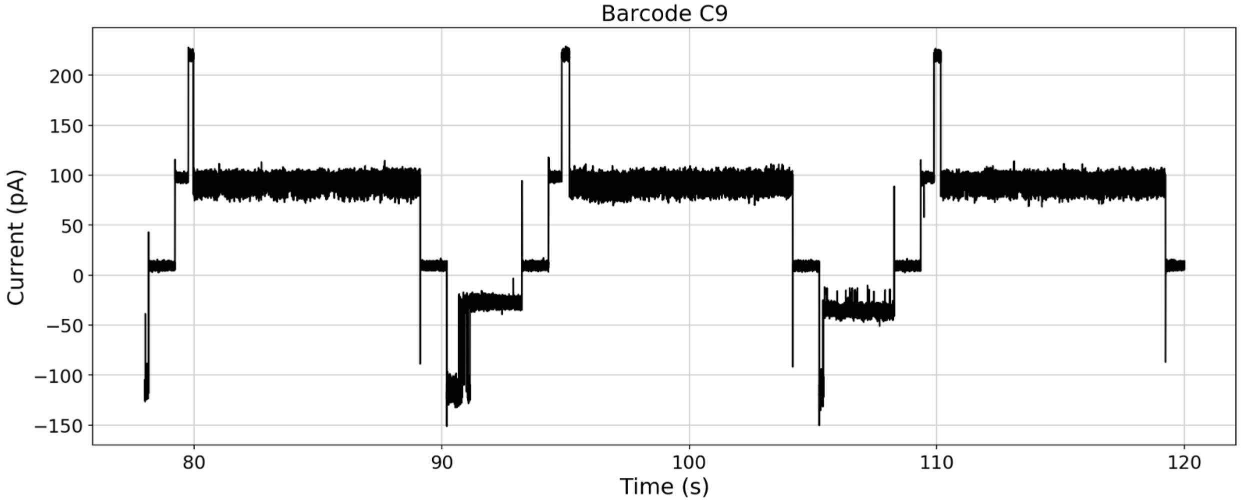
Barcode C9
Current (pA)
Time (s)

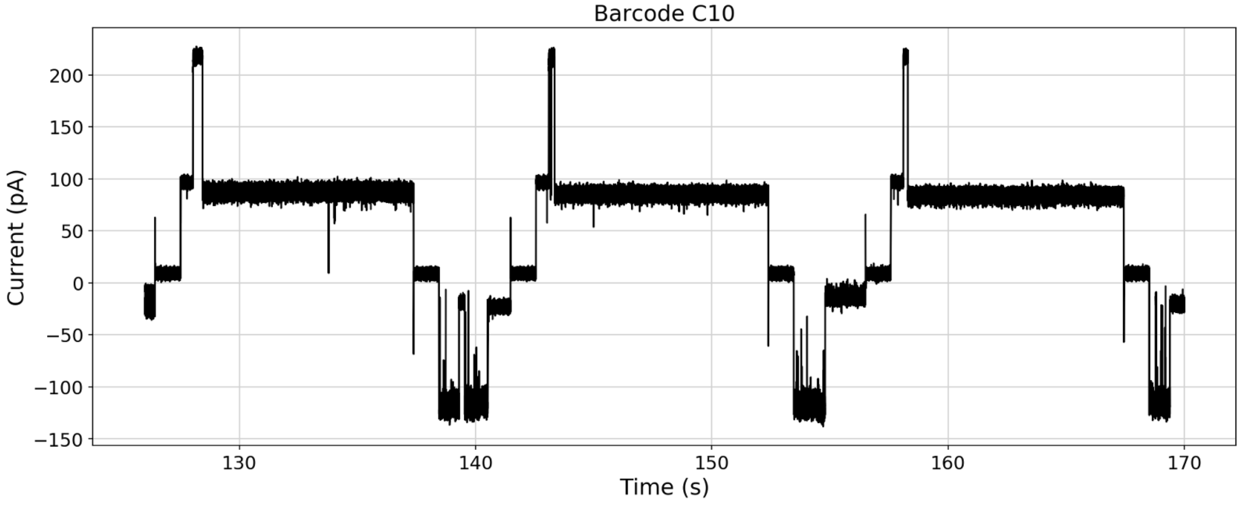
Barcode C10
Current (pA)
Time (s)

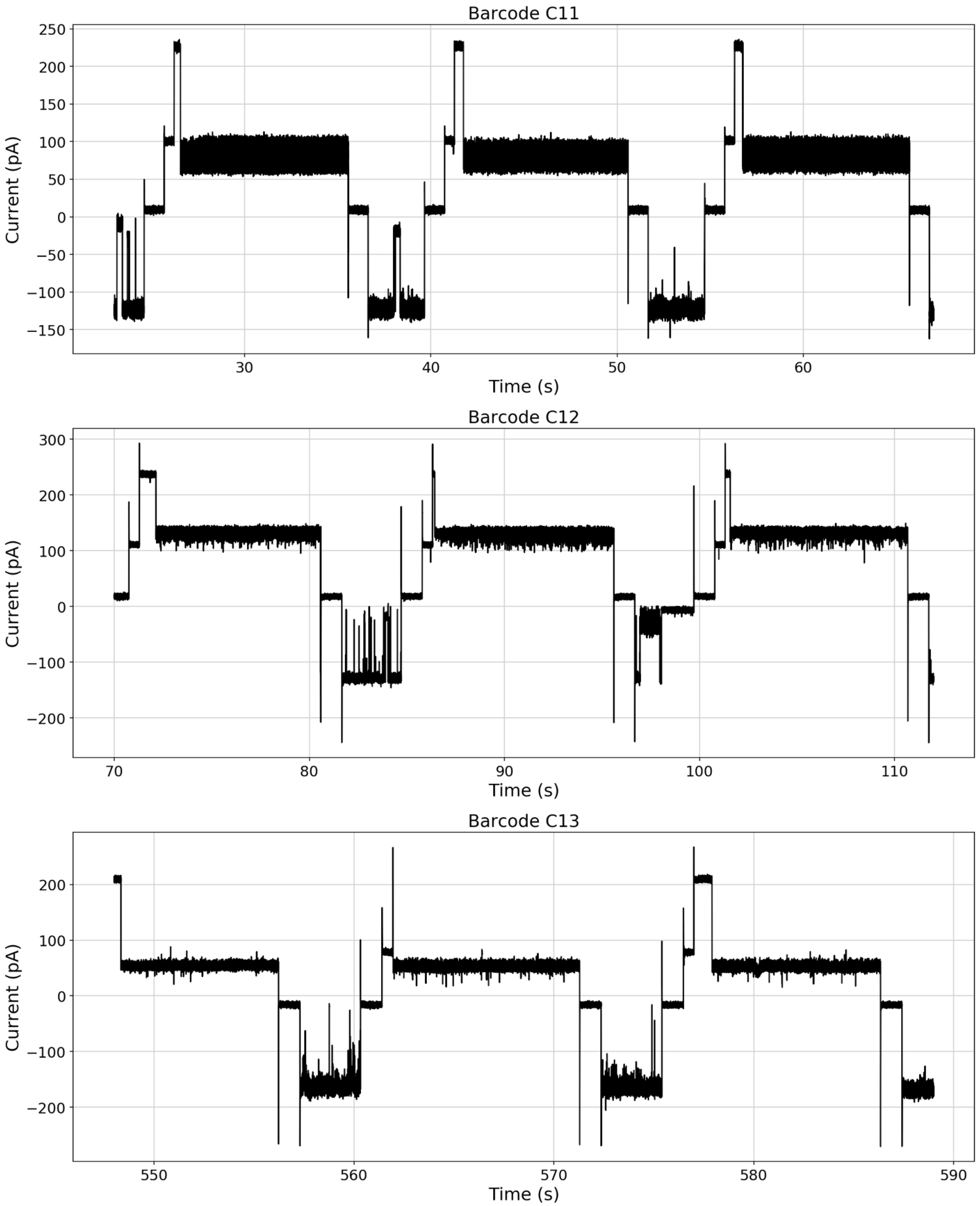

Supplementary Figure 8: Example nanopore raw traces for all barcodes.

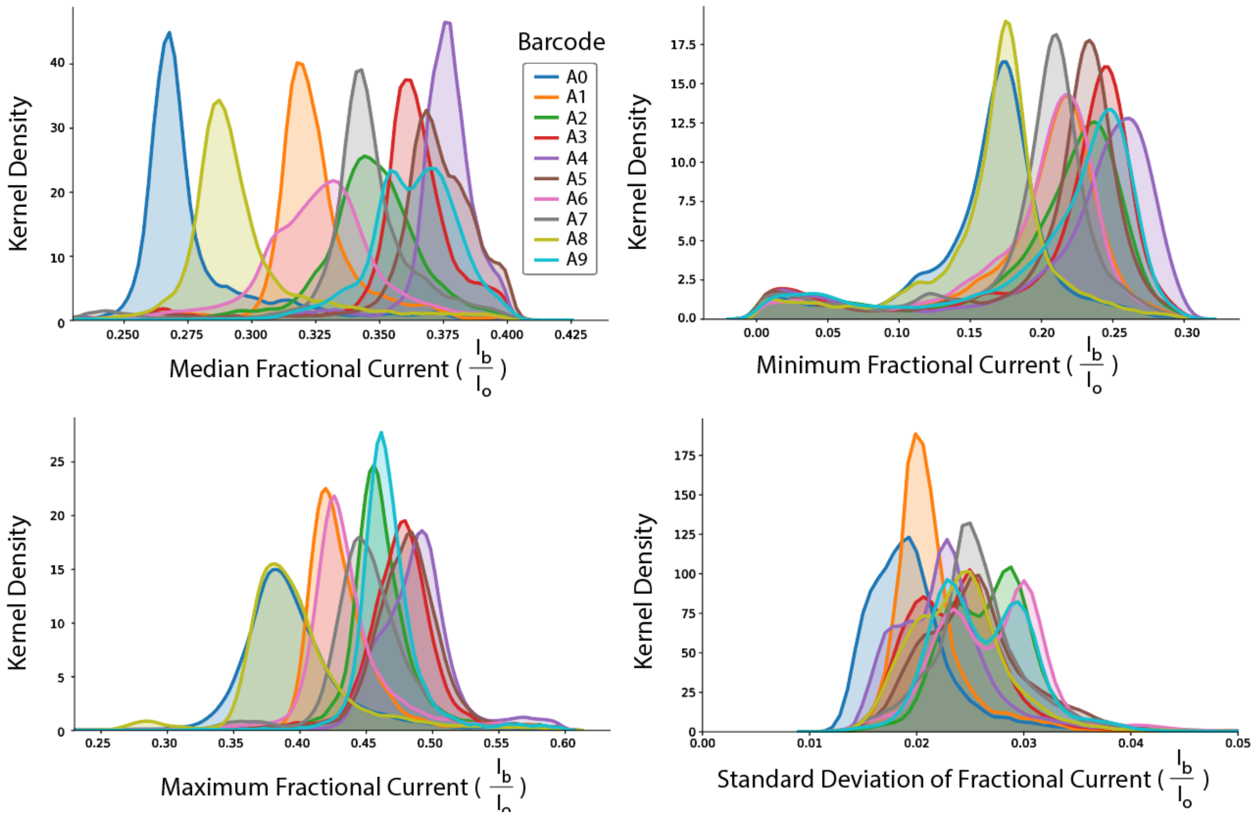

Supplementary Figure 9: Distributions of median, minimum, maximum, and standard deviation of nanopore fractional current for each barcode in Set A. Each distribution is composed of ~14500 data points.

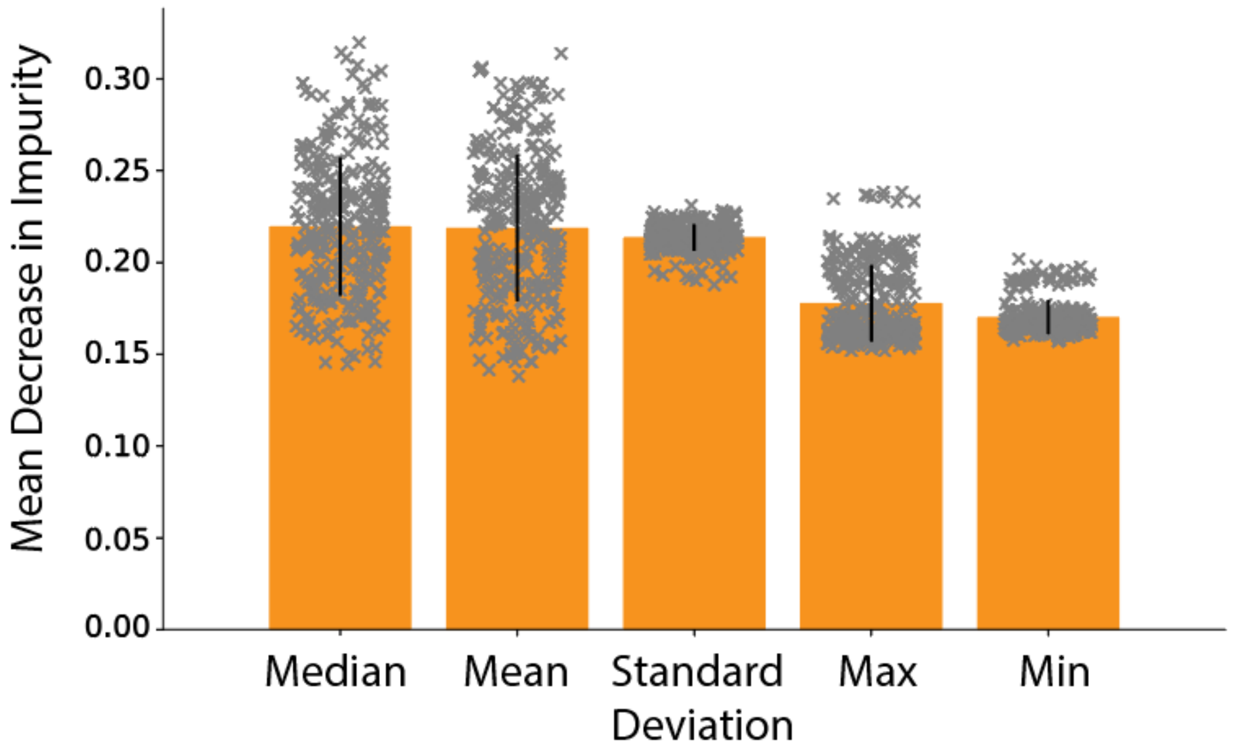

Supplementary Figure 10: Extracted feature importances (based on https://scikit-learn.org/stable/auto_examples/ensemble/plot_forest_importances.html) for a Random Forest model trained to discriminate between all ten barcodes in Set A. Bars for each feature represent mean decrease in impurity ± standard deviation from 300 datapoints (model contains 300 decision trees).

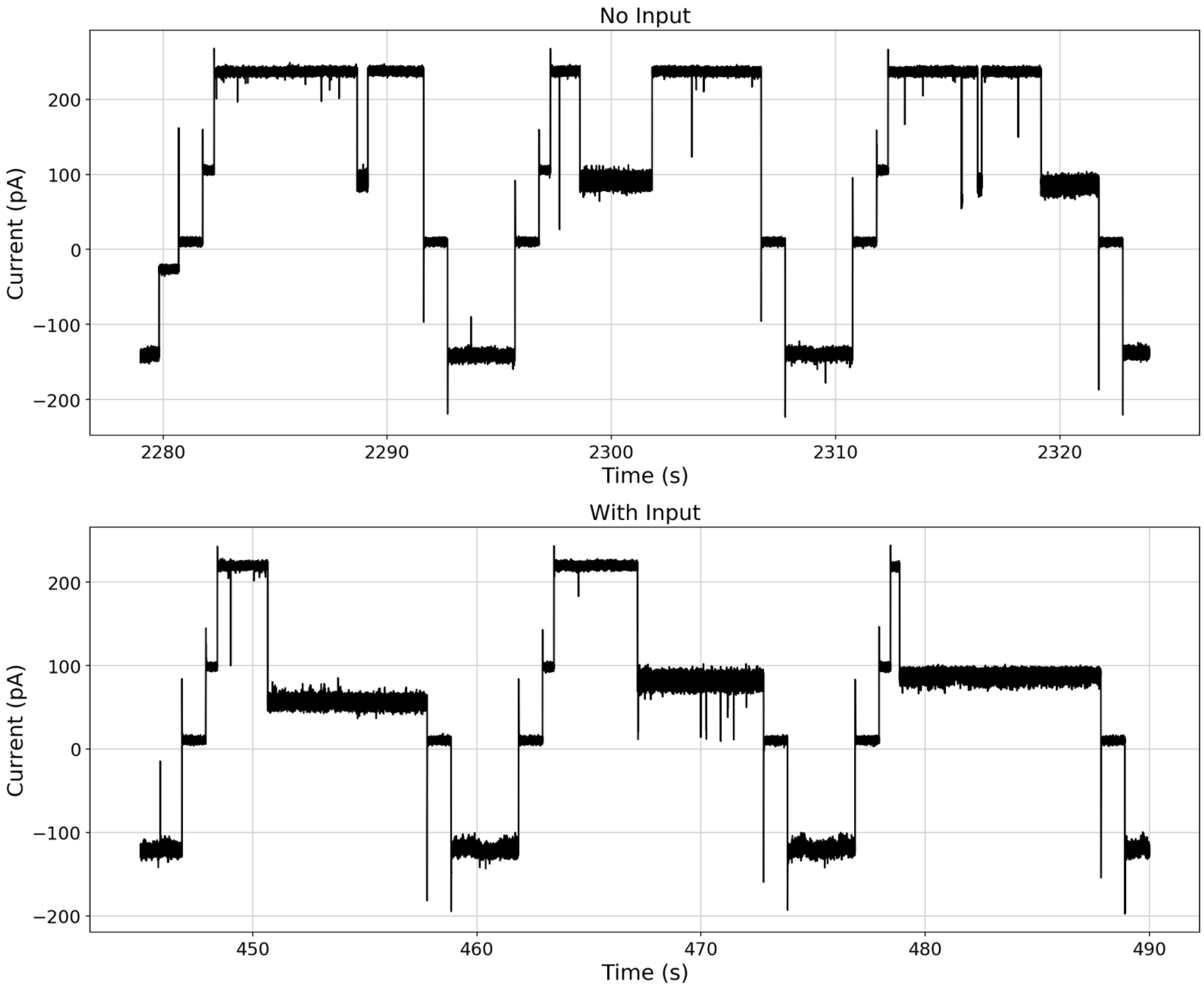

Supplementary Figure 11: Example nanopore raw traces from two-circuit multiplexed kinetics experiment in Figure 3e. Upper plot is from the No Input sample and bottom plot is from the With Input sample. Both traces were collected at least 60 min into the run.

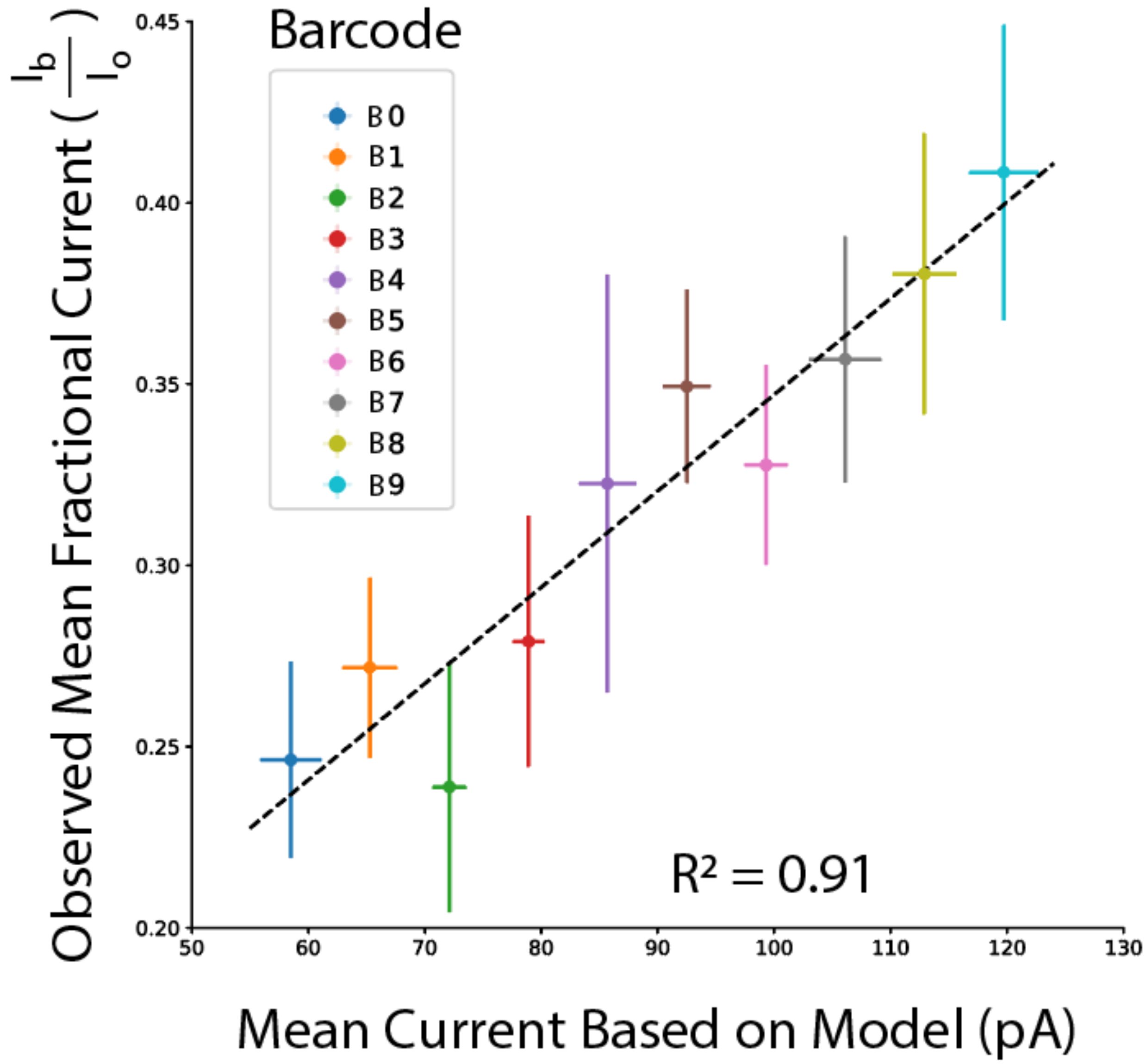

Supplementary Figure 12: Correlation of observed mean fractional currents to the mean current from predictive model for barcodes B1-B9. Y-axis position ± standard deviation for each point represents mean current from 150 nanopore capture events. X-axis position ± standard deviation for each point represents mean current as reported in ONT's kmer models (https://github.com/nanoporetech/kmer_models).

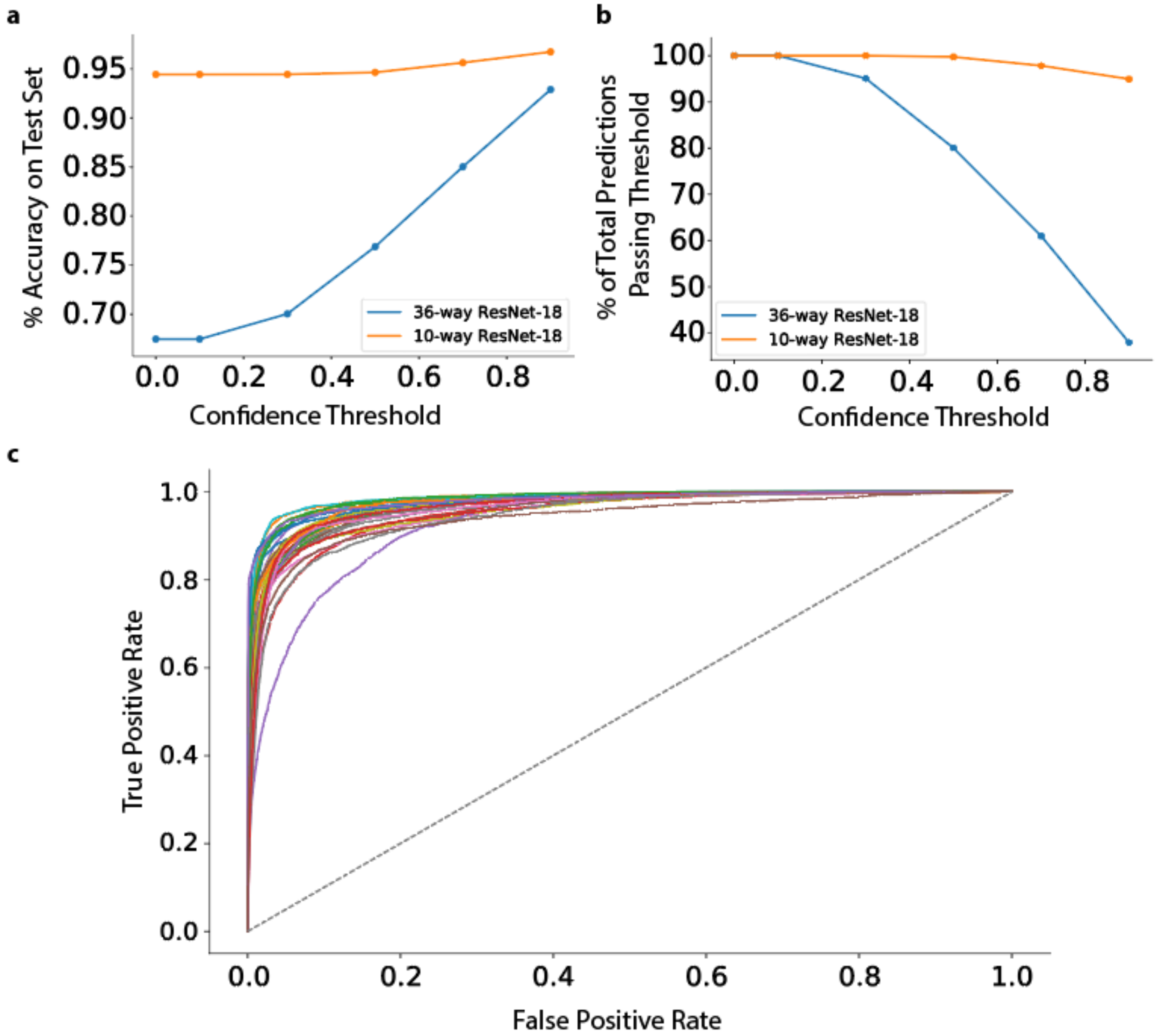

Supplementary Figure 13: Increasing the confidence threshold for a 36-class ResNet-18 and a 10-class ResNet-18 classifier **a)** boosts prediction accuracy on the withheld test set but **b)** decreases the proportion of examples that pass the threshold and contribute to that accuracy. The 36-class test set contained 2350 examples per class while the 10-class test set contained 1119 examples per class. **c)** ROC curves for each class (colored lines) of the 36-class ResNet-18 were computed using prediction scores from binary classification of each class against all other classes. Gray, dotted line represents the ROC curve of a random binary classifier. Average AUC of all classes is 0.969.

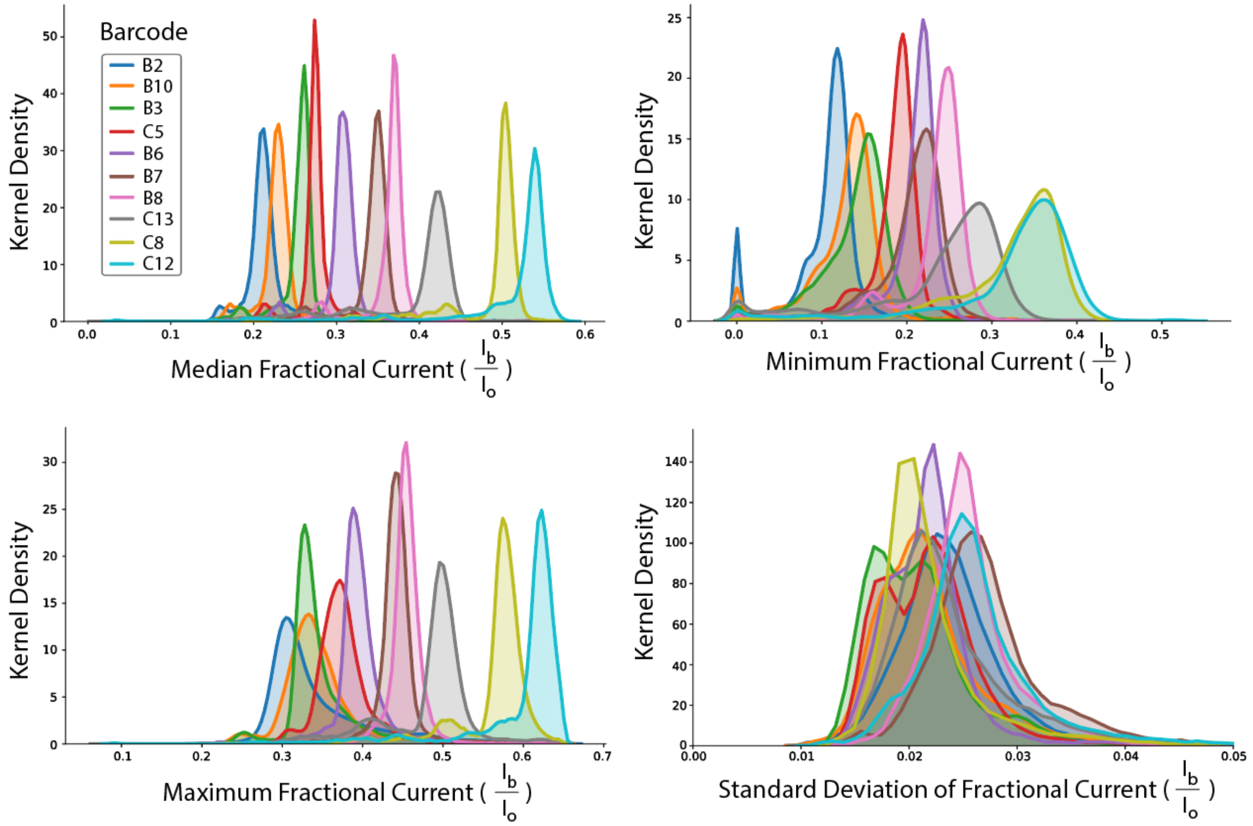

Supplementary Figure 14: Distributions of median, minimum, maximum, and standard deviation of nanopore fractional current for each barcode in the selected set of orthogonal barcodes used for multiplexing experiments. Each distribution is composed of ~13000 data points.

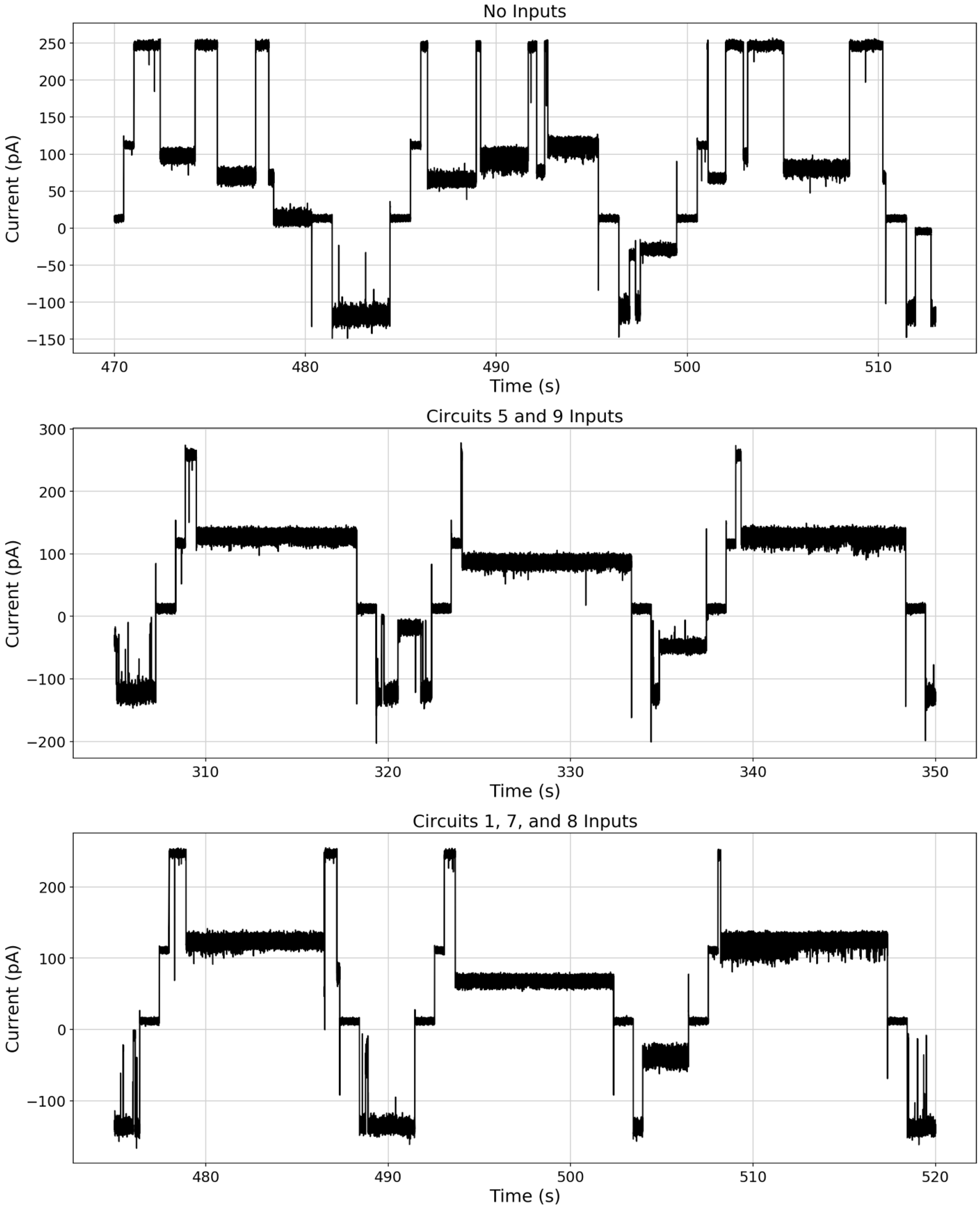

Supplementary Figure 15: Example nanopore raw traces from circuit multiplexing experiment in Figure 4d for sample with no inputs added, sample with Circuits 5 and 9 inputs added, and sample with Circuits 1, 7, and 8 inputs added.

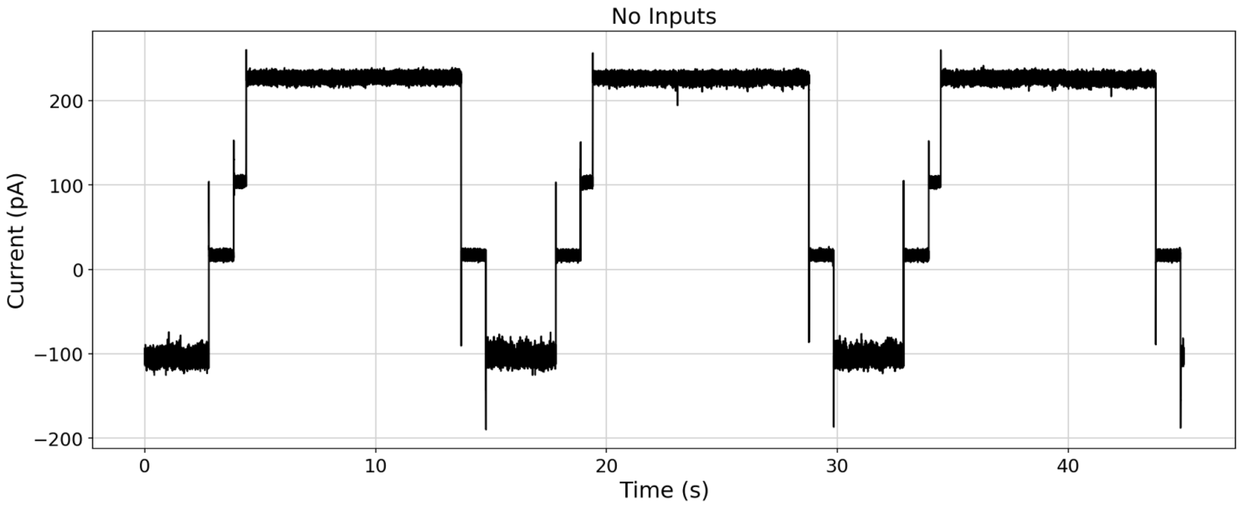
No Inputs
Current (pA)
Time (s)
200
100
0
−100
−200
0
10
20
30
40

302

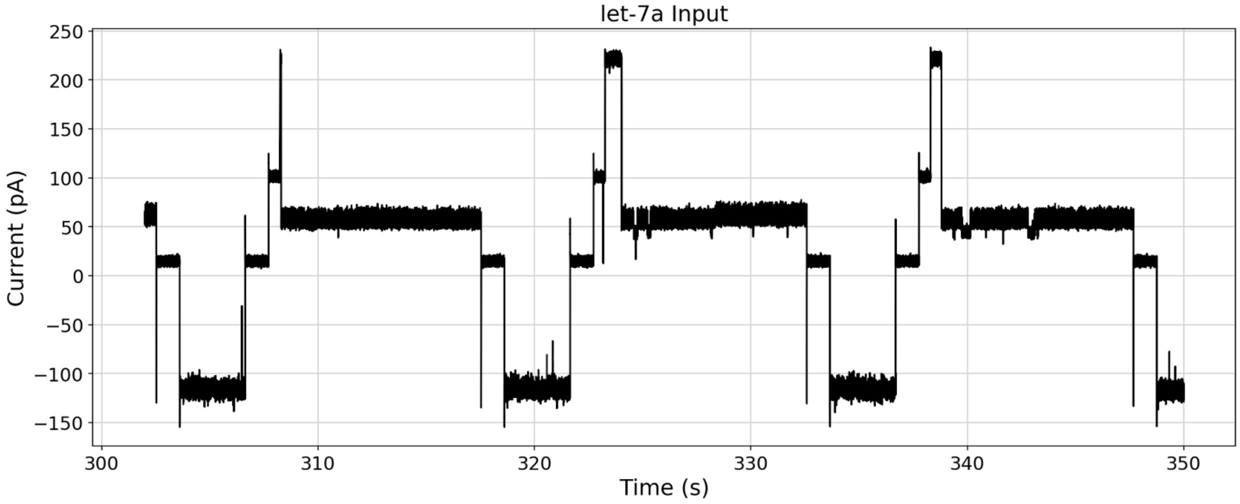
let-7a Input
Current (pA)
Time (s)
250
200
150
100
50
0
−50
−100
−150
300
310
320
330
340
350

303

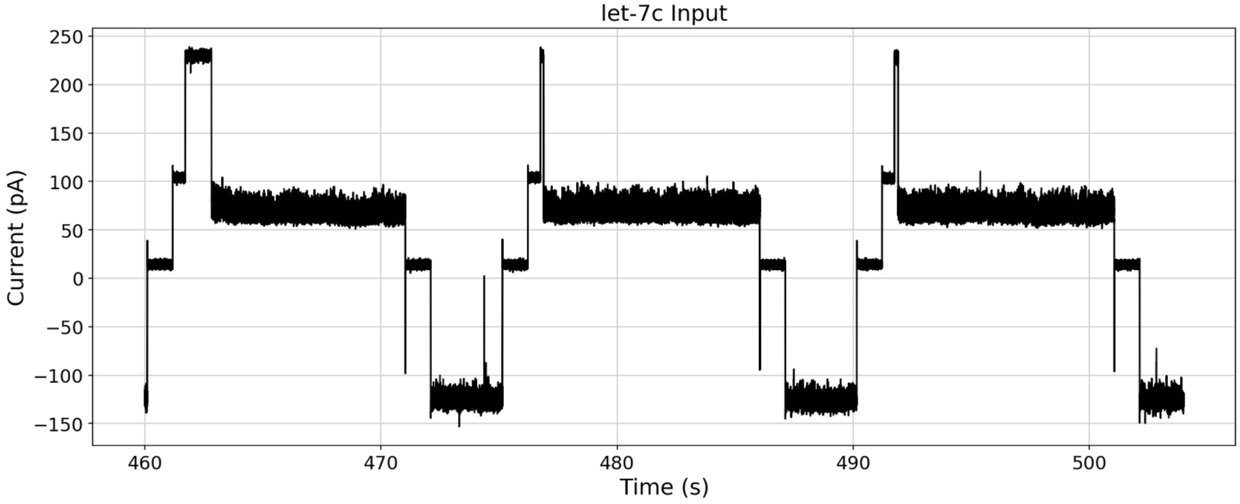
let-7c Input
Current (pA)
Time (s)
250
200
150
100
50
0
−50
−100
−150
460
470
480
490
500

304

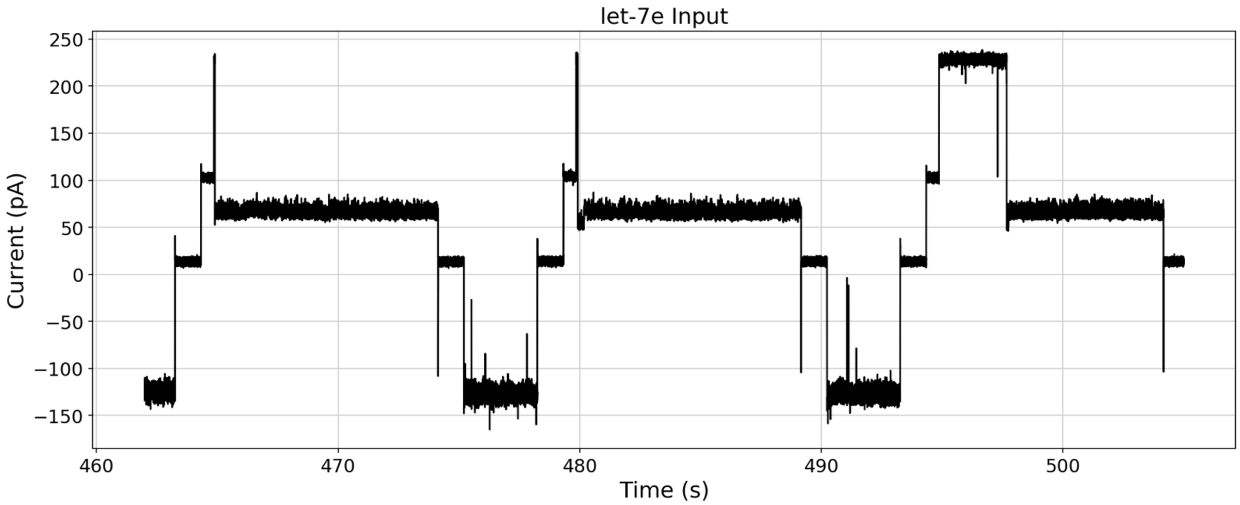
let-7e Input
Current (pA)
250
200
150
100
50
0
−50
−100
−150
460
470
480
490
500
Time (s)

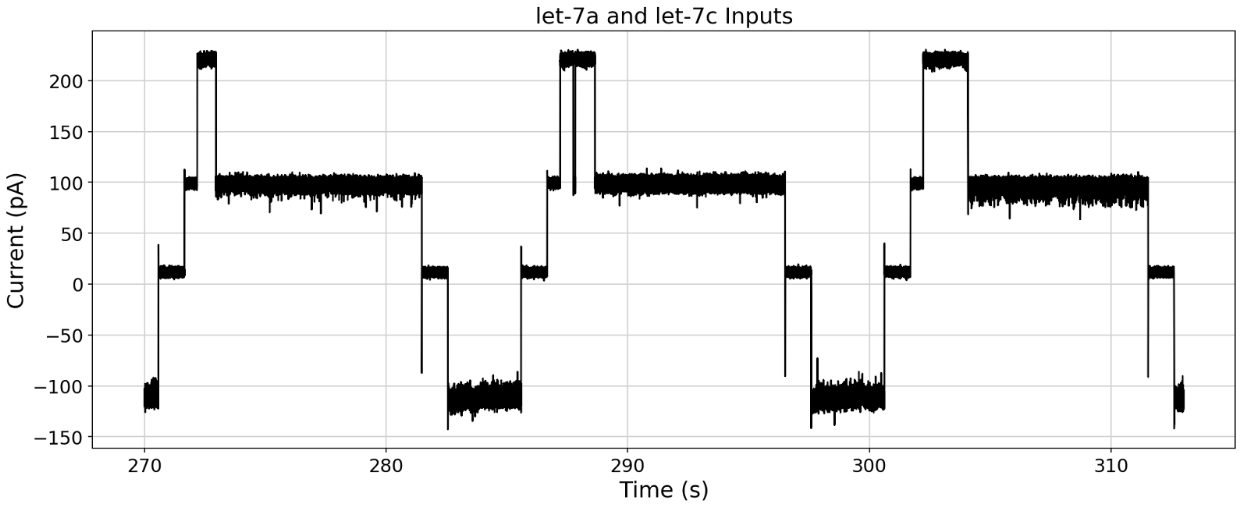
let-7a and let-7c Inputs
Current (pA)
200
150
100
50
0
−50
−100
−150
270
280
290
300
310
Time (s)

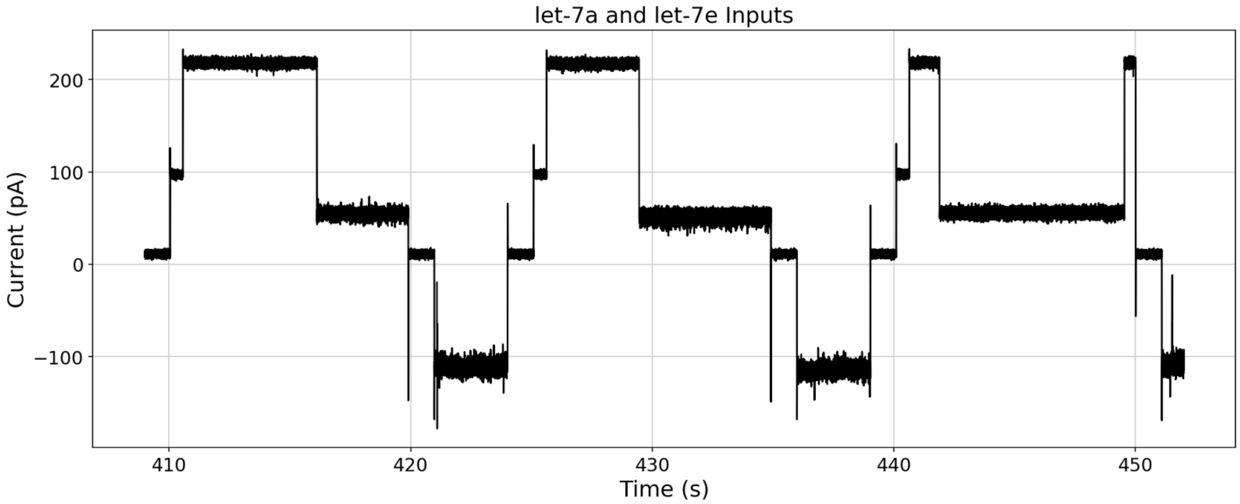
let-7a and let-7e Inputs
Current (pA)
200
100
0
−100
410
420
430
440
450
Time (s)

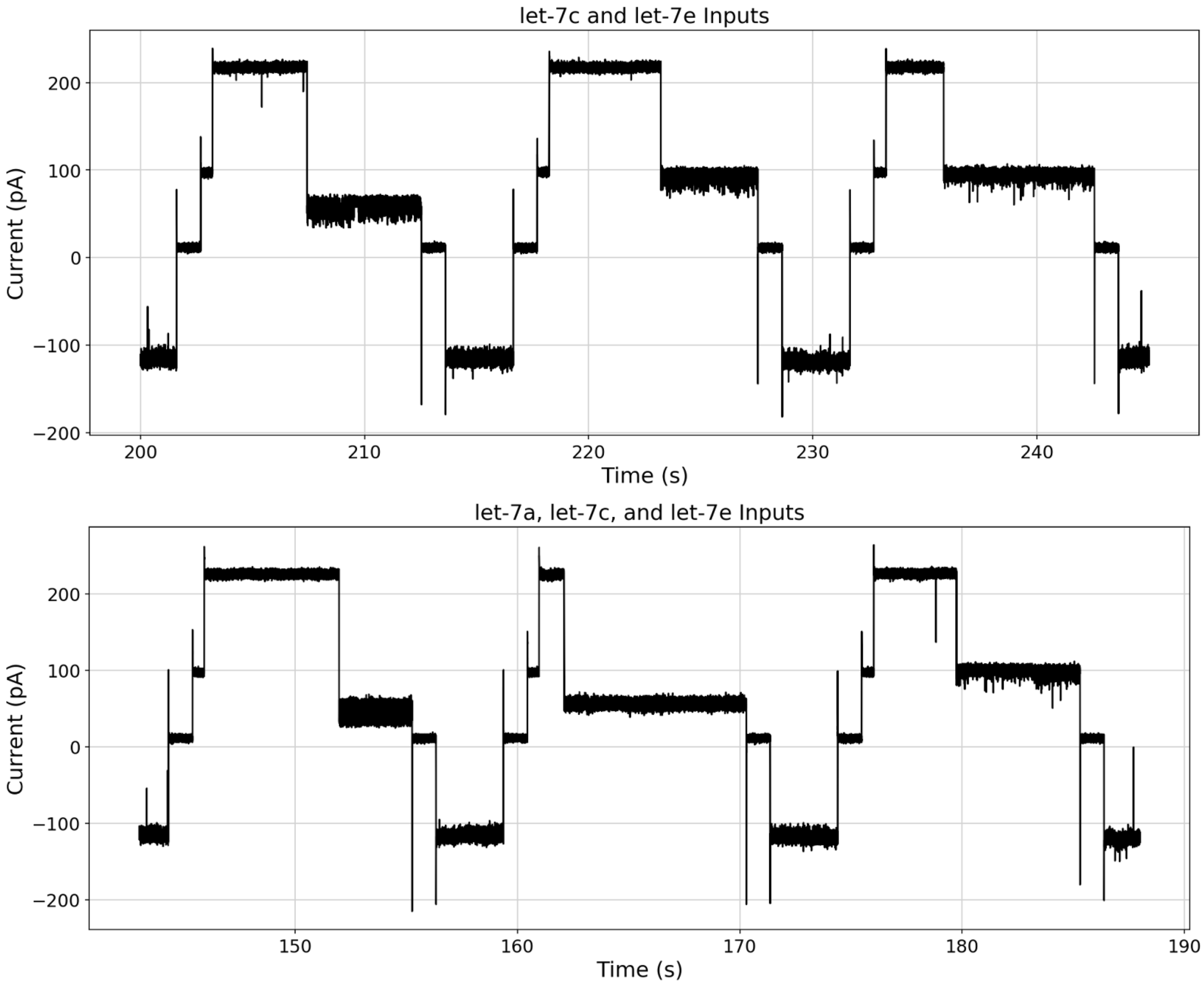

Supplementary Figure 16: Example nanopore raw traces from the let-7 miRNA detection experiment in Figure 5c. Each plot is a different sample. These samples are a no input control and every combination of let-7a, let-7c, and let-7e inputs.

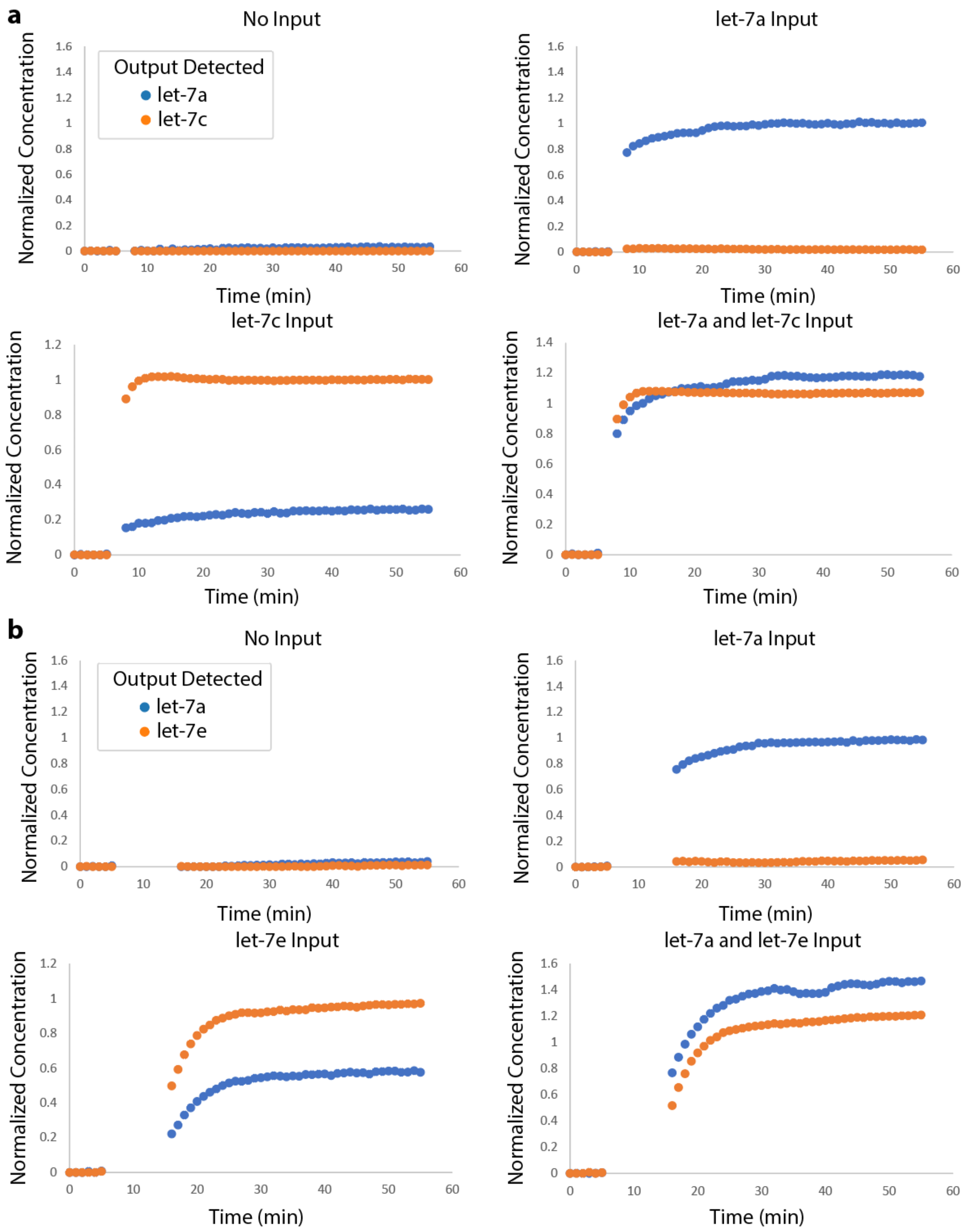

a
No Input
Output Detected
let-7a
let-7c
let-7a Input
let-7c Input
let-7a and let-7c Input
b
No Input
Output Detected
let-7a
let-7e
let-7a Input
let-7e Input
let-7a and let-7e Input
Normalized Concentration
Time (min)

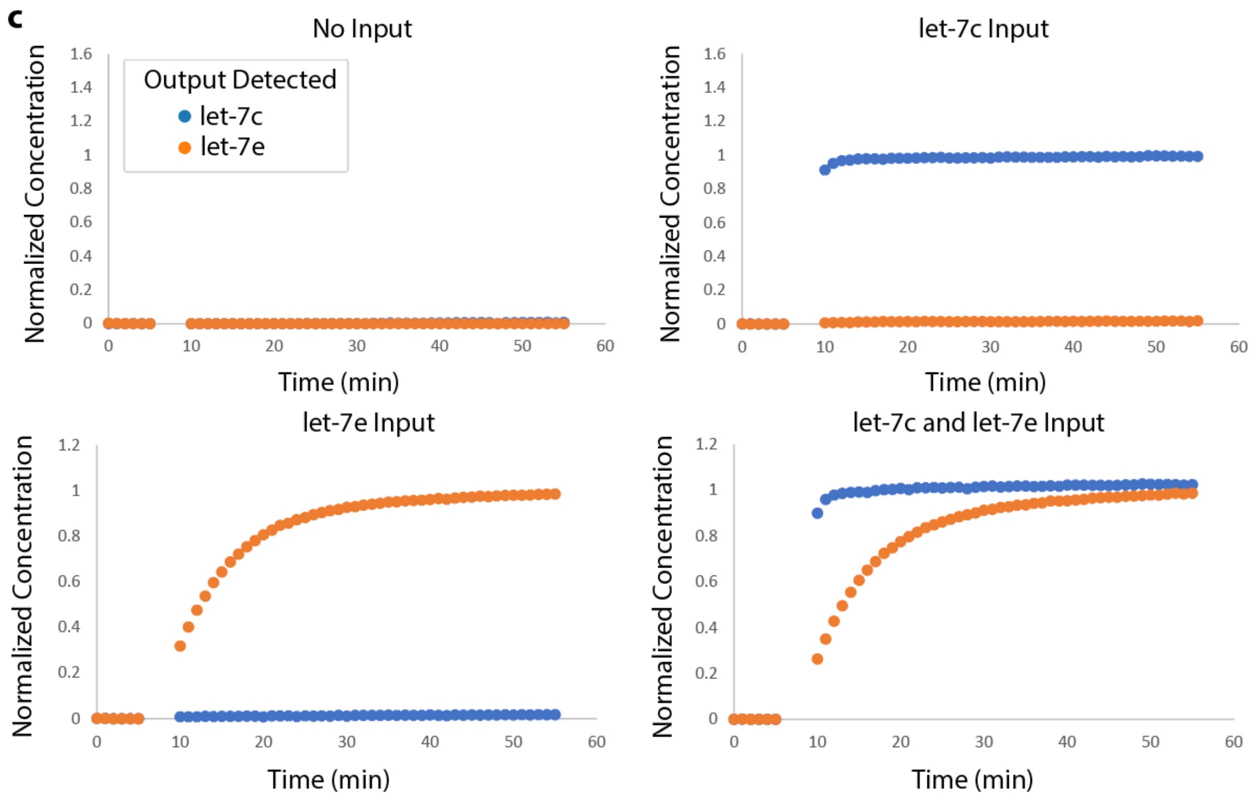

Supplementary Figure 17: Normalized concentrations of let-7 probe output measured by fluorospectrometer. **a)** let-7a and let-7c probes, **b)** let-7a and let-7e probes, and **c)** let-7c and let-7e probes were multiplexed and their response to the introduction of miRNA input strands was measured. Each probe was present at 100 nM, each helper strand at 130 nM, each input strand at 50 nM, and streptavidin at 1200 nM.

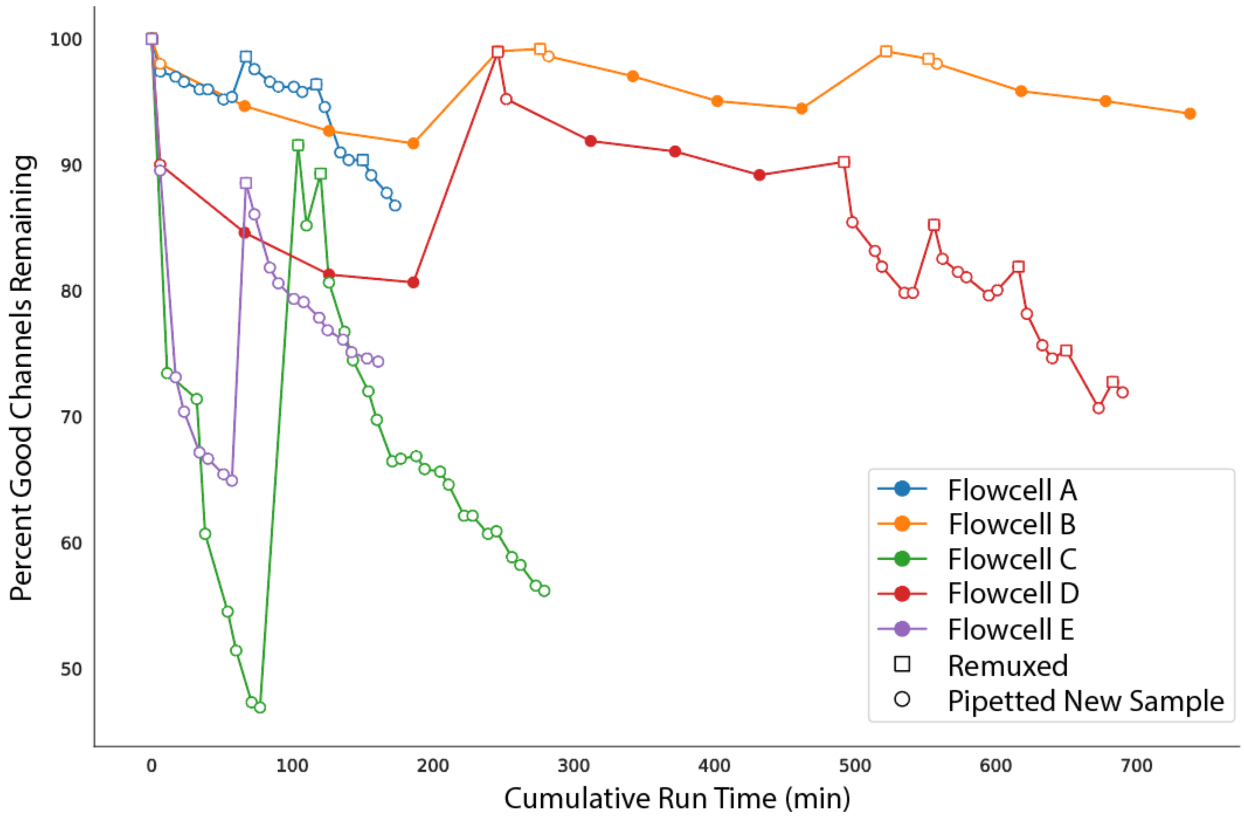

Supplementary Figure 18: Plot showing the number of good channels on MinION R9.4.1 flow cells over run time. Each flow cell (colored line) begins with 400-512 good channels. Channel loss occurs at a higher rate when new samples (circles) are pipetted into the flow cell more frequently. Remuxing (squares) of the flow cell recovers channels.

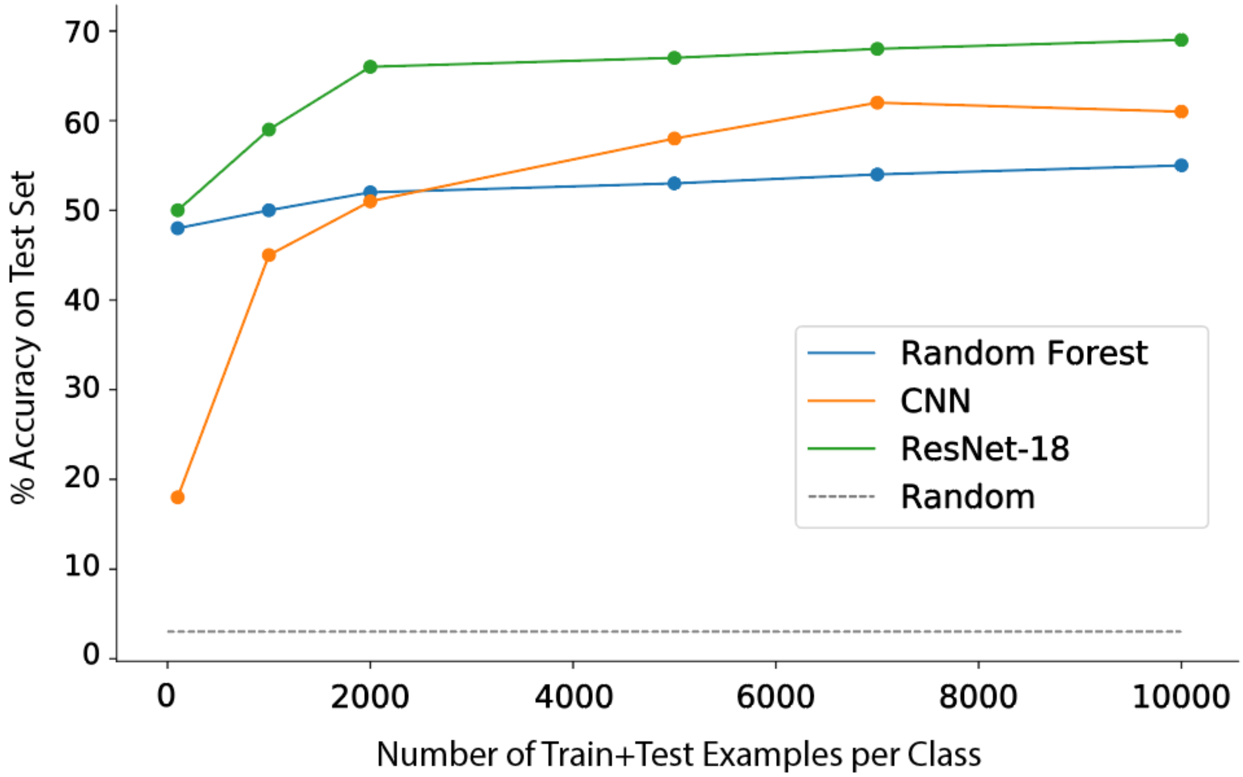

Supplementary Figure 19: Impact of training sample size on the performance of a 33-class Random Forest, 4-layer CNN (CNN), and ResNet-18 classifier, as measured by percent prediction accuracy on the withheld test set. Expected performance of a model that predicts classes completely at random is shown for reference.
