# Supplementary material for: A nanopore interface for higher bandwidth DNA computing
Source: Nat Commun. 2022 Aug 20;13:4904. doi: 10.1038/s41467-022-32526-3 (PMC9392746; doi:10.1038/s41467-022-32526-3)
Supplement: Supplementary file 2 — Reporting Summary [file 41467_2022_32526_MOESM2_ESM.pdf]

nature research

Corresponding author(s): Jeff Nivala

Last updated by author(s): 2022/07/07

Reporting Summary

Nature Research wishes to improve the reproducibility of the work that we publish. This form provides structure for consistency and transparency in reporting. For further information on Nature Research policies, see our Editorial Policies and the Editorial Policy Checklist.

Statistics

For all statistical analyses, confirm that the following items are present in the figure legend, table legend, main text, or Methods section.

n/a	Confirmed	
☒	☐	The exact sample size (*n*) for each experimental group/condition, given as a discrete number and unit of measurement
☐	☒	A statement on whether measurements were taken from distinct samples or whether the same sample was measured repeatedly
☒	☐	The statistical test(s) used AND whether they are one- or two-sided<br>*Only common tests should be described solely by name; describe more complex techniques in the Methods section.*
☒	☐	A description of all covariates tested
☒	☐	A description of any assumptions or corrections, such as tests of normality and adjustment for multiple comparisons
☐	☒	A full description of the statistical parameters including central tendency (e.g. means) or other basic estimates (e.g. regression coefficient) AND variation (e.g. standard deviation) or associated estimates of uncertainty (e.g. confidence intervals)
☒	☐	For null hypothesis testing, the test statistic (e.g. *F*, *t*, *r*) with confidence intervals, effect sizes, degrees of freedom and *P* value noted<br>*Give P values as exact values whenever suitable.*
☒	☐	For Bayesian analysis, information on the choice of priors and Markov chain Monte Carlo settings
☒	☐	For hierarchical and complex designs, identification of the appropriate level for tests and full reporting of outcomes
☒	☐	Estimates of effect sizes (e.g. Cohen's *d*, Pearson's *r*), indicating how they were calculated

Our web collection on statistics for biologists contains articles on many of the points above.

Software and code

Policy information about availability of computer code

Data collection	Custom MinION MinKNOW script enabling collection of raw current data from a nanopore sequencing run and implementation of periodic voltage flips. This custom script and research version of MinKNOW is available from Oxford Nanopore Technologies.
Data analysis	Custom scripts for extraction, filtering, classification, and quantification of nanopore capture events are available at https://github.com/uwmisl/dna-nanopore-computing.

For manuscripts utilizing custom algorithms or software that are central to the research but not yet described in published literature, software must be made available to editors and reviewers. We strongly encourage code deposition in a community repository (e.g. GitHub). See the Nature Research guidelines for submitting code & software for further information.

Data

Policy information about availability of data

All manuscripts must include a data availability statement. This statement should provide the following information, where applicable:

- Accession codes, unique identifiers, or web links for publicly available datasets
- A list of figures that have associated raw data
- A description of any restrictions on data availability

A subset of the entire dataset (Fig. 4d, Replicate 1) analyzed in this study is publicly available (DOI: 10.5281/zenodo.6846356). The entire dataset is prohibitively large to share publicly currently, however it is available from the authors on reasonable request.

Field-specific reporting

Please select the one below that is the best fit for your research. If you are not sure, read the appropriate sections before making your selection.

- [x] Life sciences
- [ ] Behavioural & social sciences
- [ ] Ecological, evolutionary & environmental sciences

For a reference copy of the document with all sections, see nature.com/documents/nr-reporting-summary-flat.pdf

Life sciences study design

All studies must disclose on these points even when the disclosure is negative.

Sample size	For each of the density plots (Fig. 2b, Fig. 3b) and ML models trained, sample size was determined by the barcode class with the lowest number of captures obtained in nanopore experiments.
Data exclusions	No data were excluded from analyses.
Replication	The standard curve in Fig. 1d comprises three replicates, each with a differently barcoded strand and all successful. The circuit kinetics experiments in Fig. 2c and Fig. 3e were performed independently with no replicates. Three biological replicates were conducted for the five-circuit multiplexing (Fig. 4d) and let-7 variant detection (Fig. 5c) experiments with all replicates being successful.
Randomization	Randomization was not relevant to our study because no experimental groups were being compared.
Blinding	Blinding was not relevant to our study because no experimental groups were being compared.

Reporting for specific materials, systems and methods

We require information from authors about some types of materials, experimental systems and methods used in many studies. Here, indicate whether each material, system or method listed is relevant to your study. If you are not sure if a list item applies to your research, read the appropriate section before selecting a response.

Materials & experimental systems

n/a	Involved in the study	
x		Antibodies
x		Eukaryotic cell lines
x		Palaeontology and archaeology
x		Animals and other organisms
x		Human research participants
x		Clinical data
x		Dual use research of concern

Methods

n/a	Involved in the study	
x		ChIP-seq
x		Flow cytometry
x		MRI-based neuroimaging
